# Novel regulation mechanism of histone methyltransferase SMYD5 in rheumatoid arthritis

**DOI:** 10.1186/s11658-025-00707-9

**Published:** 2025-03-31

**Authors:** Chenxi Xiao, Zhenghua Su, Jialin Zhao, Subei Tan, Mengting He, Yuhui Li, Jiayao Liu, Jie Xu, Yajie Hu, Zhongzheng Li, Chunxiang Fan, Xinhua Liu

**Affiliations:** 1 Department of Traditional Chinese Medicine, Shanghai Pudong Hospital, Pharmacophenomics Laboratory, Phenome Research Center of TCM, Human Phenome Institute, Fudan University, 825, Zhangheng Road, Pudong New District, Shanghai, China; 2 The 9th Hospital of Ningbo, 68, Xiangbei Road, Jiangbei District, Ningbo, 315020 Zhejiang China

**Keywords:** Rheumatoid arthritis, Fibroblast-like synoviocytes, SMYD5, FoxO1, HK2, NF-κB

## Abstract

**Background:**

Fibroblast-like synoviocytes (FLS) are crucial for maintaining synovial homeostasis. SMYD5, a member of the histone lysine methyltransferase subfamily SMYDs, is involved in many pathological processes. This study aimed to investigate the role of SMYD5 in regulating synovial fibroblast homeostasis and the pathogenesis of rheumatoid arthritis (RA).

**Methods:**

Proteomic screening was conducted to assess SMYD5 expression in the synovium of patients with osteoarthritis (OA) and RA. In vitro, interleukin-1 beta (IL-1β) was used to induce proliferation and inflammation in FLS. Further, we performed loss-of-function and gain-of-function experiments to investigate the biological function of SMYD5. In vivo, adeno-associated virus (AAV) vectors carrying SMYD5 short-hairpin RNA (AAV-sh*SMYD5*) were injected into the knee joints to knock down SMYD5 in a collagen-induced arthritis (CIA) mouse model to evaluate its role in joint damage.

**Results:**

We observed a significant elevation of SMYD5 expression in the synovial tissues of patients with RA and IL-1β-induced FLS. SMYD5 facilitated posttranslational modifications and activated downstream signaling pathways, thereby promoting proliferation and inflammation in FLS. Mechanistically, SMYD5 mediated the methylation of Forkhead box protein O1 (FoxO1), which accelerated its degradation through ubiquitination, resulting in substantial FLS proliferation. Additionally, SMYD5 promoted lactate release to activate NF-κB signaling pathways by upregulating hexokinases-2 (HK2) expression, a key glycolytic enzyme, thereby intensifying the inflammatory response in FLS. Supporting these findings, intraarticular delivery of AAV-mediated SMYD5 knockdown in the CIA mice model effectively alleviated joint swelling, bone erosion, and overall arthritis severity.

**Conclusions:**

Together, these findings suggest that SMYD5 is a dual target for regulating synovial fibroblast homeostasis and the pathogenesis of RA. Targeting SMYD5 through local treatment strategies may provide a novel therapeutic approach for RA, particularly when combined with immunotherapy.

**Graphical abstract:**

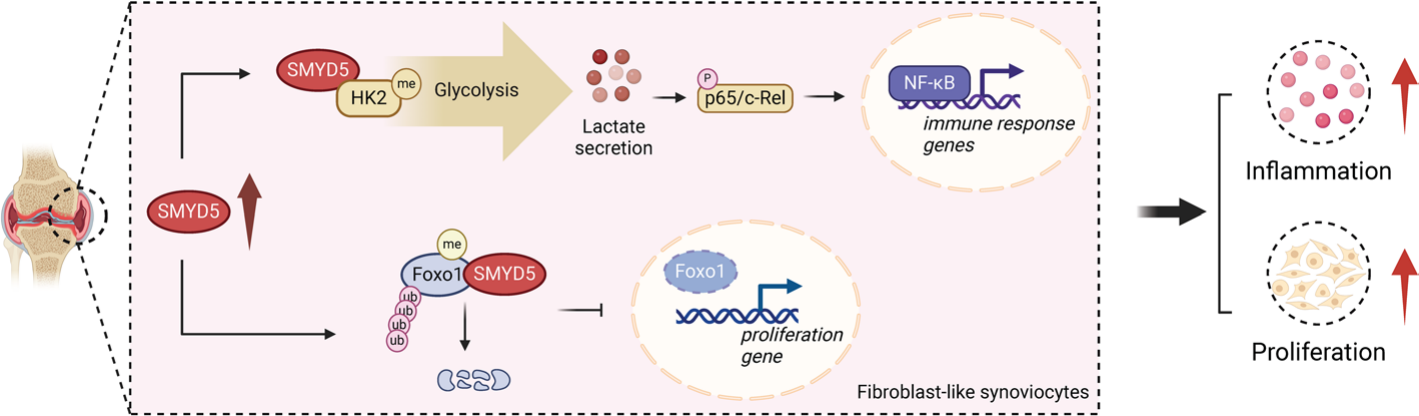

**Supplementary Information:**

The online version contains supplementary material available at 10.1186/s11658-025-00707-9.

## Introduction

Rheumatoid arthritis (RA) is a chronic autoimmune joint disease characterized by progressive bone and cartilage damage [1]. Despite advancements in targeted and immunosuppressive therapies, many patients experience persistent symptoms and poor responses to current treatments, highlighting the urgent need for new therapeutic strategies [2–4]. In recent years, the role of fibroblast-like synoviocytes (FLS) in RA pathogenesis has gained increasing attention, with growing interest in targeting FLS for potential therapeutic benefits [5–7].

FLS are specialized mesenchymal cells that regulate synovial fluid composition in healthy joints by producing lubricants such as hyaluronic acid, which nourish and lubricate the cartilage surface. Additionally, FLS contribute to the maintenance of synovial extracellular matrix (ECM) by producing components such as fibronectin, collagen, and ECM-degrading enzymes. In RA, activation of immune cells leads to excessive secretion of cytokines, including interleukin-1 (IL-1), tumor necrosis factor (TNF), and platelet-derived growth factor (PDGF). These cytokines drive the proliferation, invasion, and inflammation in the synovium, manifested by high expression of proteins related to cell proliferation (e.g., Cyclin D1, PCNA), matrix metalloproteinases (MMPs), and inflammatory factors (e.g., iNOS, COX2) in FLS. This transformation causes the synovium to form an aggressive, proliferative tissue mass known as pannus. At the pannus–cartilage interface, FLS produce high levels of MMPs, cytokines, and chemokines, which degrade the collagen-rich structure of the joints, leading to cartilage and bone destruction—key features of RA progression [5, 8]. Although research has focused on controlling FLS proliferation and inflammation, progress remains limited, highlighting the need for novel therapeutic targets and strategies.

Emerging evidence has revealed that epigenetics plays a key role in synovial proliferation and RA pathogenesis by integrating genetic and environmental factors [9, 10]. At the onset of RA, DNA hypomethylation and abnormal histone acetylation have been observed in pathways mediating FLS phenotypic changes and extracellular matrix interactions [11, 12], suggesting that targeting epigenetic alterations in FLS may offer a novel therapeutic strategy. SMYD5, a member of the SET and MYND domain-containing protein family (SMYDs), maintains nucleosomal chromatin compaction by catalyzing histone H4 lysine 20 (H4K20) methylation, thereby regulating DNA-templated processes and promoting transcriptional silencing [13]. Research on SMYD5 and its mediated H4K20 methylation mainly focuses on the self-renewal and differentiation of embryonic stem cells [14, 15]. A few studies have reported that SMYD5 plays a crucial role as an epigenetic regulator in immune-related diseases, such as inflammatory bowel disease (IBD) [13, 16]. However, its role in other autoimmune diseases, including RA, remains largely unexplored. Our previous work has shown that SMYD2, another member of the SMYDs family, mediates RA progression by regulating the TRAF2–NF-κB signaling axis [17]. Given the conserved SET catalytic domain shared by SMYD2 and SMYD5, it is worth investigating whether SMYD5 also plays a regulatory role in RA.

In this study, we observed elevated expression of SMYD5 in synovial tissues from patients with RA and IL-1β-induced FLS. Further investigation revealed that SMYD5 promoted posttranslational modifications and activated downstream signaling pathways that enhanced FLS proliferation and inflammation. Notably, SMYD5 knockdown in a collagen-induced arthritis (CIA) mouse model improved synovial repair and alleviated joint injury, suggesting that SMYD5 may serve as a potential therapeutic target for RA.

## Materials and methods

### Reagents and antibodies

Dulbecco’s modified Eagle’s medium (DMEM) and fetal bovine serum (FBS) were obtained from Gibco-BRL (Grand Island, NY, USA). Other cell culture reagents were obtained from HyClone (Logan, UT, USA) unless stated otherwise. Recombinant human IL-1β was obtained from PeproTech (Cranbury, NJ, USA). The 2-DG and AS1842856 were purchased from MedChemExpress (Monmouth Junction, NJ, USA). Antibodies were obtained from the following commercial sources: antibody against SMYD5 was obtained from Absci (Vancouver, WA, USA); antibodies against iNOS, COX2, β-actin, β-tubulin, and GAPDH were purchased from Proteintech (Wuhan, China); antibodies against p65, p-p65, p-Ikk, p-IκBα, FoxO1, and HK2 were obtained from Cell Signaling Technology (Danvers, MA, USA); antibody against Cyclin D1 was obtained from Abcam (Cambridge, MA, USA); and antibody against MMP9 was obtained from Servicebio Technology (Wuhan, China). The catalog number and usage concentration of antibodies are shown in Additional file 1: Table S1.

### Induction of CIA mice and treatment

Male DBA/1 mice (6–8 weeks of age) were purchased from SLAC Laboratory Animal (Shanghai, China) and were used to establish CIA mice model. Adeno-associated virus vectors carrying SMYD5 short-hairpin RNA (AAV-sh*SMYD5*) or negative control short-hairpin RNA (AAV-sh*Ctrl*) were constructed and produced by HanBio (Shanghai, China), and were delivered intraarticularly for arthritis prevention. DBA/1 mice were randomly divided into three groups (*n* = 8 in each group): AAV-sh*Ctrl* + sham group, AAV-sh*Ctrl* + CIA group, and AAV-sh*SMYD5* + CIA group. All mice were housed under specific pathogen-free (SPF) conditions at a stable temperature of 24 °C, and the experimental protocol conformed to the Animal Welfare Act Guide for the Use and Care of Laboratory Animals and was approved by the Institutional Animal Care and Use Committee, Fudan University, China. Only male mice were selected in our study to avoid potential interference of hormones.

The CIA mice model was established as previously described [18]. In brief, on day 0, DBA/1 mice were immunized intradermally at the base of the tail with bovine type II collagen (CII) (Chondrex, QA, USA) emulsified in Complete Freund’s adjuvant (CFA, Chondrex). On day 14, DBA/1 mice received first CII immunization were randomly chosen to receive an intraarticular injection of 10  μL AAV-sh*Ctrl* or AAV-sh*SMYD5* for SMYD5 target intervention (1 × 10^12^ vg) after anesthesia with isoflurane. Two weeks after AAV delivery, secondary immunization was performed by intradermal injection of incomplete Freund’s adjuvant (IFA, Chondrex). Joint swelling of mice was evaluated every 2 days starting from day 18 based on the Arthritis Index (see Additional file 1: Table S2). After 52 days, mice were subjected to micro-computed tomography (CT) assay and sacrificed. Knee joint tissues were harvested for subsequent experiments.

### Cell isolation, culture, and treatment

The rat primary FLS were isolated from synovium tissues of rats as described previously [19]. In brief, synovium tissues were minced and digested with 2 mg/mL type II collagenase (Worthington, NJ, USA) for 2 h at 37 °C. Afterward, 2 mL of trypsin was added, and the incubation continued until the tissue was fully digested. The digested tissues were filtered through 100-μm filters. After centrifugation at 1200 rpm for 5 min, cells were resuspended and cultured in DMEM containing 10% FBS and 1% penicillin/streptomycin in a 5% CO_2_ humidified atmosphere at 37 °C. IL-1β (10 ng/mL, 24 h) was used to stimulate FLS proliferation and inflammation in vitro.

### Micro-CT

Each mouse was positioned on a scanning table in sequence. The left and right knee joint regions were scanned separately using a high-speed in vivo micro-CT scanner (Quantum GX, PerkinElmer, Waltham, MA, USA). After scanning, three-dimensional images were reconstructed.

### Histological analysis of synovium

Knee joint tissues were fixed in 4% paraformaldehyde and embedded in paraffin to make 8-µm-thick paraffin sections. After deparaffinization, tissue sections were stained with hematoxylin and eosin (H&E), Safranin O-Fast Green or Toluidine Blue O to assess joint inflammation and cartilage damage. Histological scores were assigned based on representative H&E or Toluidine Blue and Safranin O images to quantify synovial inflammation and cartilage depletion in each group as described previously (see Additional file 1: Table S3) [20].

### Immunohistochemistry (IHC)

Paraffin-embedded joint tissue sections were deparaffinized and rehydrated in xylene and graded ethanol in turn. Then antigen retrieval was performed by heating the sections at 96 °C for 15 min in improved citrate antigen retrieval solution (Beyotime, Shanghai, China) in a water bath. Following retrieval, immunohistochemistry was carried out using the mouse- and rabbit-specific horseradish peroxidase (HRP)/3,3′-diaminobenzidine (DAB) (ABC) Detection IHC Kit (Abcam). In brief, the sections were placed in hydrogen peroxide block for 10 min to inhibit endogenous peroxidase activity, followed by protein block for 5 min to prevent nonspecific background staining. The sections were then incubated with primary antibodies including anti-Cyclin D1, anti-PCNA or anti-SMYD5 overnight at 4 °C. The next day, the sections were reacted with the biotinylated secondary antibody and HRP-labeled-streptavidin for proper time at room temperature. DAB was used to visualize antibody binding, and hematoxylin was used to label nuclei. The images were captured using a Zeiss microscope.

### Small interfering RNA (siRNA) transfection

Rat *SMYD5*, *FoxO1*, *HK2* siRNA and control siRNA were produced by GenePharma (Shanghai, China). Cells with 30–50% confluence were transfected with siRNA for 24 h via Lipofectamine RNAiMax reagent (ThermoFisher Scientific, Waltham, MA, USA)) following the recommendations of the manufacturer, and then cells were cultured for an additional 48 h with fresh serum DMEM medium. The knockdown efficiency of SMYD5 was verified by Western blot. The sequences of relevant siRNA are listed in Additional file 1: Table S4.

### Plasmid transfection

pCMV-6 × His-SMYD5(human)-Neo (His-SMYD5), pCDNA3.1-FOXO1(human)-3 × HA-SV40-Neo (HA-FoxO1), and pCMV-3 × FLAG-HK2(human)-Neo (Flag-HK2) plasmids were purchased from Miaolingbio (Wuhan, China). HiPure Plasmid EF Maxi Kit (Magen, Guangzhou, China) was used to amplify these plasmids. HEK293T cells with 80–90% confluence were cotransfected with indicated plasmids for 24 h using Lipofectamine 2000 (ThermoFisher Scientific) according to the manufacturer’s instructions, and then cultured for an additional 48 h with fresh serum DMEM medium or performed other treatments. Cells were harvested 72 h after transfection.

### Lentivirus generation and infection

Rat HA-SMYD5 overexpression lentivirus vector (pCMV-Smyd5 (rat)-HA-Neo) was purchased from Miaolingbio (Wuhan, China). SMYD5 expression plasmid, recombinant plasmid, and packaging vectors (psPAX2 and PMD2.G) were cotransfected into HEK293T cells using Lipofectamine 2000 (ThermoFisher Scientific) according to the manufacturer’s instructions. The virus supernatant was collected at 48 and 72 h after transfection. After removing cell debris using a 0.45-μm filter, the virus supernatant was mixed with complete culture medium in 1:1 ratio and added into the culture medium of FLS together with polybrene. After 24 h, the medium was replaced with DMEM containing 10% FBS. Cells were harvested 72 h after transfection to verify SMYD5 expression or perform other tests.

### Nuclear and cytoplasmic extraction

FLS were harvested by trypsin–ethylenediaminetetraacetic acid (EDTA) and then centrifuged at 500*g* for 5 min. The cytoplasmic and nuclear components were separated and extracted using NE-PER Nuclear and Cytoplasmic Extraction Kit (ThermoFisher Scientific) in accordance with the manufacturer’s instructions.

### Cycloheximide chase analysis

FLS were pretreated as described in the text and corresponding figure legends prior to treatment with 50 μg/ml cycloheximide (CHX) for the indicated time. Cells were then lysed for Western blot analysis.

### RNA isolation and quantitative real-time polymerase chain reaction (RT-qPCR)

Total RNA was extracted from FLS by Trizol Reagent (Takara, Japan). Total RNA (2 μg) from each sample was reverse-transcribed into cDNA by using AdvanceFast 1st Strand cDNA Synthesis Kit (Yeasen, Shanghai, China) following the manufacturer’s instructions. qPCR was performed using Universal Blue qPCR SYBR Green Master Mix (Yeasen) and normalized relative to β-actin expression. The primer sequences used are listed in Additional file 1: Table S5.

### Western blotting

Cultured cells were lysed in ice-cold radioimmunoprecipitation assay (RIPA) buffer (Beyotime) supplemented with protease inhibitor (APExBIO, Houston, TX, USA) and phosphatase inhibitor (APExBIO). Protein quantification was performed using the Pierce™ BCA Protein Assay Kit (23227, ThermoFisher Scientific). Whole lysate samples were separated by sodium dodecyl sulfate–polyacrylamide gel electrophoresis (SDS-PAGE) and then transferred to the nitrocellulose membranes (Millipore, Burlington, MA, USA). After being blocked by 5% nonfat dried milk for 2 h, membranes were incubated with the specified primary antibodies overnight at 4 °C and combined with HRP-conjugated goat anti-rabbit or goat anti-mouse antibodies (Jackson ImmunoResearch Inc.) for another 2 h at room temperature. The images were then visualized using ChemiDoc+ (Bio-RAD, Hercules, CA, USA) after incubation with ECL (ShareBio, Shanghai, China). Signal intensity was detected and quantified by ImageJ software.

### Cell proliferation assay

Cell proliferation assay was performed by using the BeyoClick™ EdU Cell Proliferation Kit with Alexa Fluor 488 (Beyotime). Briefly, FLS were seeded on coverslips placed in 24-well plates. After different treatments, cells were labeled with 10 μM EdU for 2 h. After fixation with 4% paraformaldehyde and permeation through 0.3% Triton X-100, cells were incubated with Click Reaction Mixture for 30 min at room temperature in the dark environment, followed by staining the nuclei with DAPI. The number of EdU-positive cells was observed using fluorescence microscopy and quantified via ImageJ software.

### Cytokine analysis

The concentration of IL-6 and TNF-α in cell supernatant or mice blood samples were measured via enzyme-linked immunosorbent assay (ELISA) kits (Dakewe, Shanghai, China) according to the manufacturer’s instructions.

### Immunofluorescence staining

FLS were seeded on coverslips placed in 24-well plates. After different treatments, the cells were fixed with 4% paraformaldehyde for 30 min, permeabilized with 0.25% Triton X-100 for 10 min, and then blocked with 10% goat serum for 30 min at room temperature. The cells were then incubated with p65 primary antibody at 4 °C overnight. The following day, the cells were treated with Alexa Fluor 488 donkey anti-rabbit IgG (ThermoFisher Scientific) for 1.5 h at room temperature in the dark. DAPI was used to stain and locate nuclei. The images were observed by a fluorescence microscope (LSM780, Carl Zeiss).

### Peptide preparation for MS analysis

Synovium tissues from patients with osteoarthritis (OA) or rheumatoid arthritis (RA) were collected in accordance with the guidelines of the Ningbo Ninth Hospital (approval no. 2024LIW20). The clinical characteristics and treatment history of the patients with OA and RA are summarized in Additional file 1: Table S6. Synovium tissues were lysed in 50 mM ammonium bicarbonate (ABC) buffer, heated at 95 °C for 30 min, and digested with trypsin at 37 °C for 16 h (enzyme-to-protein ratio 1:50). Peptides were dried at 60 °C with SpeedVac (Eppendorf), dissolved in 0.1% formic acid (FA), and desalted using 3 M C18 columns. The effluent was dried in a vacuum drier at 60 °C and stored at −80 °C until liquid chromatography tandem mass spectrometry (LC–MS/MS) analysis.

### LC–MS/MS proteome analysis

For the proteome profiling samples, peptides were analyzed on a Q Exactive HF-X mass spectrometer (ThermoFisher Scientific) coupled with a high-performance liquid chromatography system (EASY nLC 1200, ThermoFisher Scientific). Dried peptide samples redissolved in solvent A (0.1% formic acid in water) were loaded onto a 2-cm self-packed trap column (100 μm inner diameter, 3 μm ReproSil-Pur C18-AQ beads, Dr. Maisch GmbH) using solvent A and separated on a 150-μm-inner-diameter column with a length of 30 cm (1.9 μm ReproSil-Pur C18-AQ beads, Dr. Maisch GmbH) over a 150-min gradient (solvent A: 0.1% formic acid in water; solvent B: 0.1% formic acid in 80% ACN) at a constant flow rate of 600 nL/min (0–150 min, 0 min, 4% B; 0–10 min, 4–13% B; 10–120 min, 13–28% B; 120–140 min, 28–50% B; 140–145 min, 50–100% B; 145–150 min, 100% B). Eluted peptides were ionized at 2 kV and introduced into the mass spectrometer. Mass spectrometry was performed in data-dependent acquisition mode. For the MS1 spectra full scan, ions with *m*/*z* ranging from 300 to 1400 were acquired by an Orbitrap mass analyzer at a high resolution of 120,000. The automatic gain control (AGC) target value was set to 3 × 10^6^. The maximal ion injection time was 80 ms. MS2 spectral acquisition was performed in the ion trap in a rapid speed mode. Precursor ions were selected and fragmented with higher energy collision dissociation (HCD) with a normalized collision energy of 27%. Fragment ions were analyzed by an ion trap mass analyzer with an AGC target at 5 × 10^4^. The maximal ion injection time of MS2 was 20 ms. Peptides that triggered MS/MS scans were dynamically excluded from further MS/MS scans for 12 s. MS raw files were processed with Firmiana (a one-stop proteomic cloud platform) [21] against the human National Center for Biotechnology Information (NCBI) RefSeq protein database (updated on 4/7/2013, 32,015 entries) using Mascot 2.4 (Matrix Science Inc., London, UK). The maximum number of missed cleavages was set to two. For the quality control of protein identification, the target-decoy-based strategy was applied to confirm that the false discovery rate (FDR) of both peptides and proteins was lower than 1%. The program percolator was used to obtain the probability value (^q^ value) and showed that the FDR (measured by the decoy hits) of every peptide–spectrum match (PSM) was lower than 1%. Differential expression of proteins associated with histone methyltransferases was analyzed and visualized in a heat map.

### Co-immunoprecipitation (co-IP)

The interactions of SMYD5 with other proteins were determined by co-IP. After transfection with His-SMYD5, HA-FoxO1, or Flag-HK2, HEK293T cells were lysed in ice-cold IP-RIPA buffer (Beyotime) supplemented with 1% protease inhibitor and 1% phenylmethylsulfonyl fluoride (PMSF) for 30 min and centrifuged at 12,000*g* at 4 °C for 10 min, then the supernatant was transferred to a new EP tube and incubated with Protein A/G PLUS-Agarose (Santa Cruz Biotechnology) for 30 min to reduce nonspecific binding. After another centrifugation step, equal amounts of lysates (500–1000 μg) were immunoprecipitated with appropriate amounts of primary antibodies (IgG antibody as control) at 4 °C on a shaker overnight, then incubated with 20 μL Protein A/G PLUS-Agarose at 4 °C for another 2 h to precipitate the immune complexes. The immunoprecipitates were collected by centrifugation at 2500 rpm for 5 min at 4 °C. The pellet was then washed three times with 1 mL ice-cold PBS buffer, and each time the centrifugation step above was repeated. After final wash, supernatant was discarded and pellet was resuspended in 60 μL of 2 × electrophoresis sample buffer (Invitrogen). The samples were then subjected to SDS-PAGE and immunoblotted with the corresponding antibodies.

### IP-MS

To identify proteins interacting with SMYD5, FLS expressing HA-SMYD5 were harvested and lysed in IP-RIPA buffer (Beyotime) containing PMSF and a protease inhibitor cocktail. We used anti-HA mouse mAbs to immunoprecipitated SMYD5 protein, and then obtained immunoprecipitation complexes using Protein A/G PLUS-Agarose as described in the “Co-immunoprecipitation” section. The immunoprecipitates were separated by SDS-PAGE, and Coomassie Brilliant Blue R250 staining was used to visualize the proteins on the gel. The HA-SMYD5 band and a nearby region (~42 kd) were excised and subjected to mass spectrometry to identify proteins interacting with SMYD5.

### In vitro methyltransferase assay

HA-SMYD5, HA-FoxO1, and Flag-HK2 recombinant proteins were purified using HA-tag or Flag-tag Protein IP Assay Kit with agarose gel (P2206S or P2202S, Beyotime). FoxO1 or HK2 recombinant protein was incubated with *S*-adenosylmethionine (10619ES02, Yeasen) and SMYD5 recombinant protein in a mixture of methylase buffer (50 mM Tris–HCl pH 8.0, 0.01%Tween-20, 1 mM 1,4-dithiothreitol (DTT), 5 mM MgCl_2_) for 1 h at 37 °C. The electrophoresis samples were then prepared and subjected to SDS-PAGE.

### In vivo ubiquitination assay

HEK293T cells were cotransfected with indicated plasmids for 24 h using Lipofectamine 2000 (ThermoFisher Scientific). Cells were treated with 10 μM MG132 for 6 h before harvest. After completing the cultivation, cells were resuspended in IP-RIPA buffer (Beyotime). The protocol continued as described in the “Co-immunoprecipitation” section. Finally, samples were subjected to immunoblotting with anti-ubiquitin antibodies.

### Statistical analysis

Results are expressed as mean ± standard error on the mean (SEM). All data analyses were performed using GraphPad Prism software. Differences between mean values of multiple groups were analyzed by one-way analysis of variance (ANOVA), and when comparing two groups using unpaired Student’s *t* test. Statistical significance was considered as *p* ≤ 0.05.

## Results

### Prominent expression of SMYD5 in synovial tissues of patients with RA and IL-1β-induced FLS

**Fig. 1:**
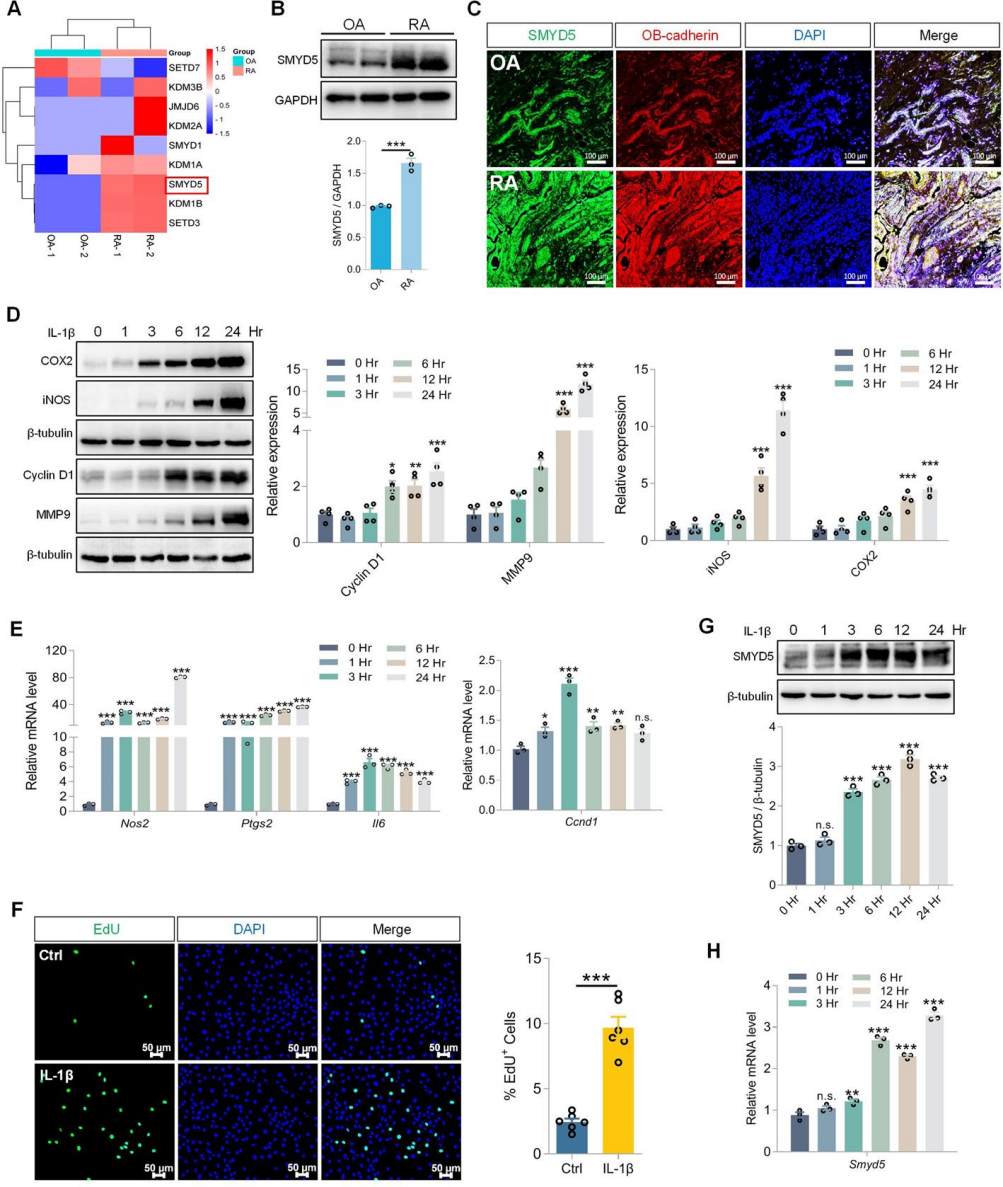
Prominent expression of SMYD5 in synovial tissues of patients with RA and IL-1β treated FLS. **A** Heatmap showing the protein expression of histone methyltransferases in synovial tissues from patients with osteoarthritis (OA) and rheumatoid arthritis (RA). **B** Immunoblot analysis and quantification of SMYD5 in synovial tissues from patients with OA or RA, *n* = 3. **C** Immunofluorescence detection of SMYD5 in synovial tissues from patients with OA or RA. Scale bars, 100 μm. **D**–**H** Rat fibroblast-like synoviocytes (FLS) were treated with IL-1β (10 ng/ml) for indicated times. **D** Immunoblot analysis and quantification of inflammation markers (iNOS and COX2) and proliferation markers (Cyclin D1 and MMP9), *n* = 4. **E** mRNA levels of *Nos2*, *Ptgs2*, *Il6*, and *Ccnd1* measured by RT-qPCR, *n* = 3. **F** Representative images of EdU staining in IL-1β treated or untreated FLS. Scale bars, 50 μm. Quantification of EdU-positive cells in each group is shown in the right panel, *n* = 6. The protein expression (**G**) and mRNA level (**H**) of SMYD5 were detected by immunoblots and RT-qPCR, respectively, *n* = 3. Quantification of SMYD5 protein expression in **G** normalized to β-tubulin. Data presented as mean ± SEM, *p* values calculated by two-tailed Student’s *t*-test (**B**, **F**) or one-way ANOVA test (**D**, **E**, **G**, **H**), *p < 0.05, **p < 0.01, ***p < 0.001, n.s. means no significance

To gain systemic insights into the epigenetic changes that occur in the synovium of patients with RA, we conducted proteomic screening on synovium from patients with OA and RA. Through proteomics screening, we found that SMYD5 expression was dramatically elevated in the synovial tissues of patients with RA compared with that of patients with OA (Fig. 1A), which was further confirmed by both Western blot (Fig. 1B) and immunofluorescence (IF) staining (Fig. 1C). In the joints of patients with RA, immune cells excessively secrete IL-1β and other cytokines, which promote the proliferation and inflammation response in FLS, playing an important role in the pathogenesis and progression of RA. To model this immune environment, we treated primary rat FLS with IL-1β, simulating the disease condition in synovial tissues. Phenotypic changes in FLS were confirmed by the upregulation of proliferation-related proteins (e.g., Cyclin D1 and MMP9) and inflammatory mediators (e.g., iNOS and COX2) in a time-dependent manner (Fig. 1D). Similarly, the transcription levels of pro-inflammatory genes such as *Nos2, Ptsgs2* and *Il6* and proliferation marker *Ccnd1* were both increased in response to IL-1β treatment as detected by RT-qPCR analysis (Fig. 1E). The abnormal proliferation phenotype of FLS mentioned above was also confirmed by EdU staining, manifested as an increase in the number of cells stained EdU-positive after IL-1β stimulation (Fig. 1F). Consistent with observations in RA synovial tissues, we found that IL-1β treatment led to SMYD5 upregulation in both protein level and mRNA level in FLS (Fig. 1G, H). Considering the synchronous changes of SMYD5 and abnormal FLS phenotype after IL-1β treatment, we speculate that SMYD5 might be an important regulatory factor for synovial injury in RA.

### SMYD5 modulates FLS proliferation in response to IL-1β

**Fig. 2:**
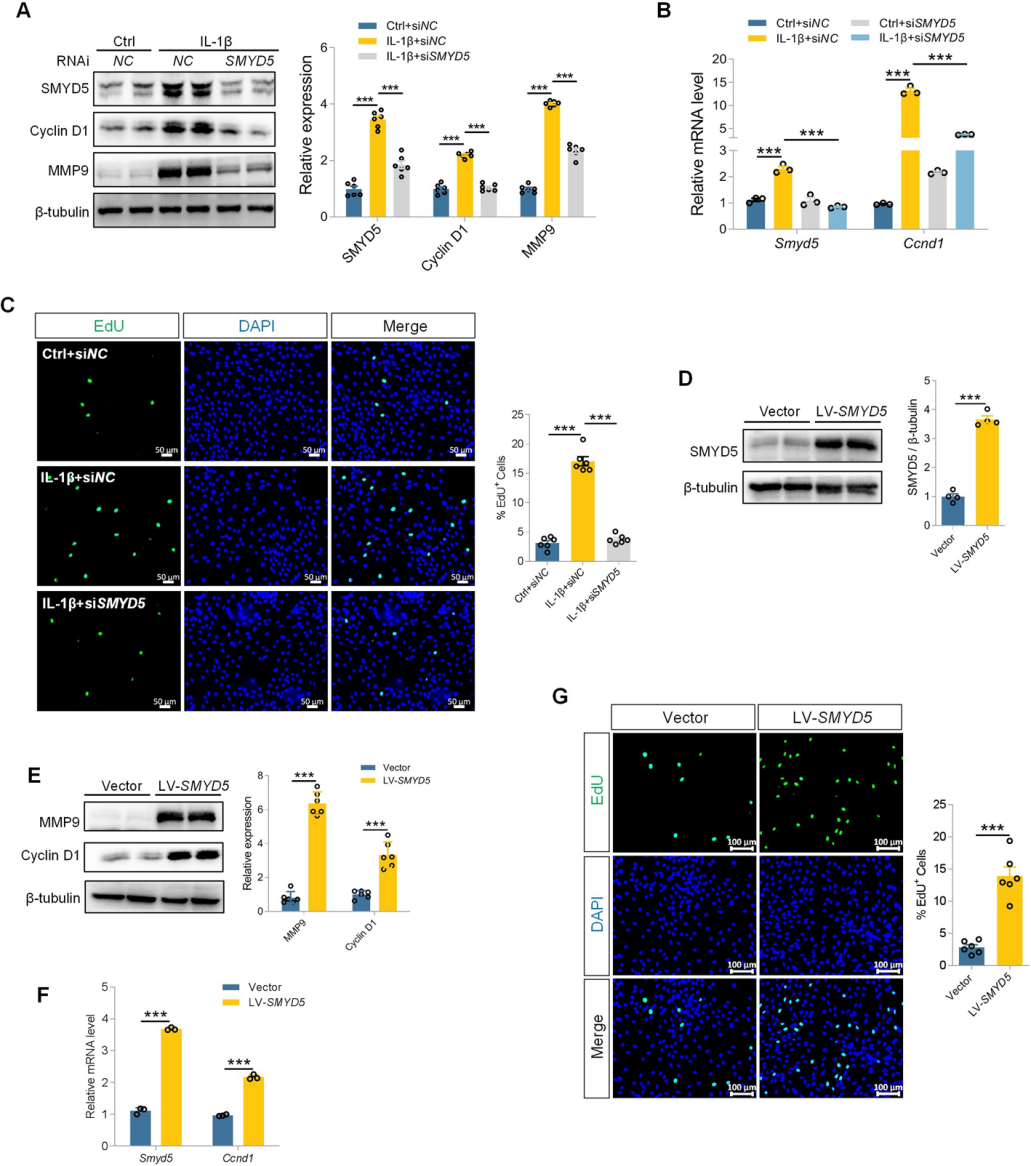
SMYD5 modulates FLS proliferation response to IL-1β. **A-C** SMYD5 was knocked down using *SMYD5* siRNA in IL-1β (10 ng/ml, 24 h) induced FLS, with control siRNA (si*NC*) as a negative control. **A** Immunoblot analysis and quantification of SMYD5, Cyclin D1, and MMP9, with β-tubulin used as a loading control, *n* = 6. **B** *Smyd5* and *Ccnd1* mRNA levels were detected by RT-qPCR analysis, *n* = 3. **C** Representative images of EdU staining in IL-1β-induced FLS pre-transfected with control or *SMYD5* siRNA. Scale bars, 50 μm. Right panel shows statistical analysis of the proportion of EdU-positive cells, *n* = 6. **D**–**F** FLS were infected with lentivirus vectors encoding SMYD5 (LV-*SMYD5*) to promote SMYD5 overexpression. **D** Immunoblot analysis and quantification of SMYD5, *n* = 4. **E** Immunoblot analysis and quantification of Cyclin D1 and MMP9, with β-tubulin used as a loading control, *n* = 6. **F** RT-qPCR analysis of *Smyd5* and *Ccnd1* mRNA levels, *n* = 3. **G** Representative images of EdU staining in LV-*SMYD5*-treated or untreated FLS. Scale bars, 100 μm. Right panel shows the quantification of EdU-positive cells, *n* = 6. Data presented as mean ± SEM, *p* values calculated by two-tailed Student’s *t*-test (**D**–**G**) or one-way ANOVA test (**A**–**C**), ^***^p < 0.001

To further explore the regulatory role of SMYD5 in FLS proliferation phenotype, we used small interfering RNA targeting SMYD5 (si*SMYD5*) to silence its expression. Compared with IL-1β challenge, SMYD5 knockdown showed a remarkable reduction in Cyclin D1 and MMP9 expression (Fig. 2A), which was further confirmed by RT-qPCR at the mRNA level (Fig. 2B). In agreement with these findings, the increased EdU incorporation during DNA synthesis in IL-1β-induced FLS was abrogated by SMYD5 knockdown (Fig. 2C). The above results indicated that silencing the SMYD5 gene could inhibit FLS proliferation phenotype induced by IL-1β. Next, we aimed to determine whether overexpression of SMYD5 alone can promote FLS proliferation in the absence of IL-1β. To do this, we used lentivirus vectors encoding SMYD5 (LV-*SMYD5*) to infect FLS. Compared with the control group, LV-*SMYD5* efficiently increased SMYD5 expression at both the protein and mRNA levels, accompanied by upregulation of proliferation marker Cyclin D1 and MMP9 expression (Fig. 2D, E). *Ccnd1* transcription was also activated after SMYD5 overexpression in FLS without IL-1β treatment (Fig. 2F). Notably, LV-*SMYD5* significantly increased the incorporation of EdU into DNA (Fig. 2G). These findings strongly support a significant positive correlation between SMYD5 and FLS proliferation.

### SMYD5 induces FoxO1 degradation through methylation modification, which promotes FLS proliferation

**Fig. 3:**
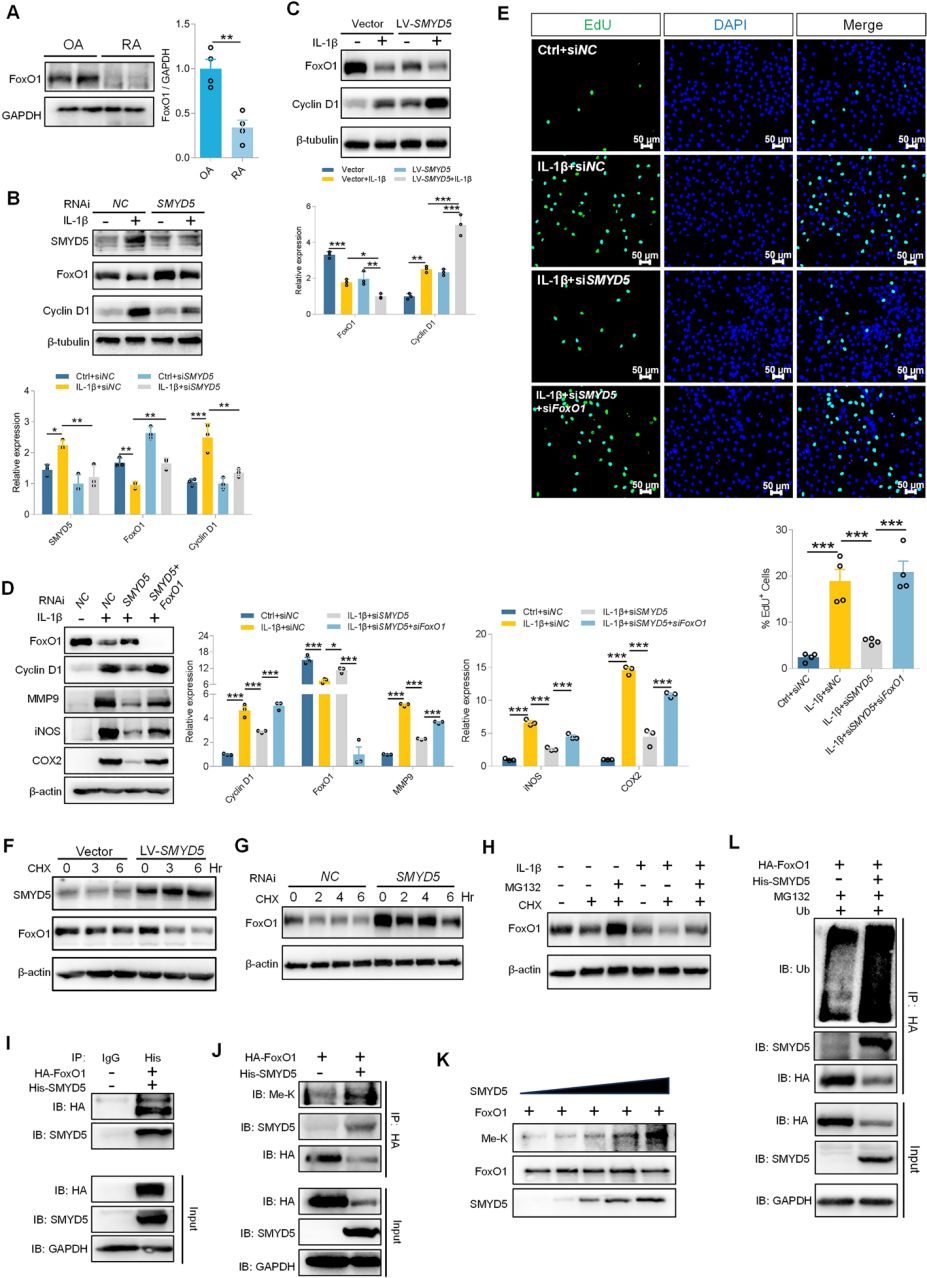
SMYD5 causes FoxO1 degradation through methylation modification, which promotes FLS proliferation. **A** Immunoblot analysis and quantification of FoxO1 in synovial tissues from patients with RA or OA, with GAPDH used as a loading control, *n* = 4. **B** FLS transfected with control or *SMYD5* siRNA were incubated with or without IL-1β; immunoblot and quantification of SMYD5, FoxO1, and Cyclin D1 are shown, using β-tubulin as a loading control, *n* = 3. **C** FLS infected with vector or LV-*SMYD5* were treated with or without IL-1β. Immunoblots for FoxO1 and Cyclin D1 were quantified relative to β-tubulin, *n* = 3. **D** FLS were transfected with *SMYD5* siRNA or cotransfected with *SMYD5* siRNA and *FoxO1* siRNA, followed by IL-1β treatment. Immunoblot analysis of FoxO1, Cyclin D1, MMP9, iNOS, and COX2 was performed, with β-actin as a control, *n* = 3. **E** EdU staining and analysis of EdU-positive cells were conducted (scale bars, 50 μm, *n* = 4). **F**, **G** FLS with SMYD5 overexpression (**F**) or silencing (**G**) were treated with CHX (50 μg/ml) and harvested at the indicated times for FoxO1 immunoblotting, β-actin as a control. **H** IL-1β-induced FLS were treated with MG132 (20 μM) and/or CHX (50 μg/ml) for 6 h. FoxO1 expression were assessed by immunoblot, using β-actin as a loading control. **I**, **J** HEK293T cells were transfected with the indicated plasmids and then immunoprecipitated with anti-His (**I**) or anti-HA (**J**) antibodies. Target proteins were immunoblotted with anti-HA, anti-SMYD5 and anti-methyl lysine antibodies. **K** In vitro methyltransferase assays. Recombinant FoxO1 was incubated with recombinant SMYD5, and reactions were analyzed by SDS-PAGE. **L** HEK293T cells were transfected with the indicated plasmids, and a co-IP assay was performed to examine FoxO1 polyubiquitylation in the presence of SMYD5. Data presented as mean ± SEM, *p* values calculated by two-tailed Student’s *t*-test (**A**) or one-way ANOVA test (**B**–**E**), *p < 0.05, **p < 0.01, ***p < 0.001

It has been well established that Forkhead box protein O1 (FoxO1), a member of the forkhead transcription factor family, negatively modulates FLS cell proliferation and survival in RA [22]. Here, we compared FoxO1 expression in the joints of patients with RA and OA. Consistent with a previous report, FoxO1 level was significantly reduced in the joints of patients with RA (Fig. 3A). By using *FoxO1* siRNA or selective inhibitor (AS1842856) to treat FLS, we confirmed the fact that knockdown or inhibition of FoxO1 could promote FLS proliferation (Fig. S1A–D). Surprisingly, we discovered that IL-1β treatment decreased FoxO1 expression, and this decrease was recovered by knocking down SMYD5, accompanied by inhibition of IL-1β-induced FLS proliferation, as demonstrated by Cyclin D1 expression (Fig. 3B). In contrast, SMYD5 overexpression downregulated FoxO1 level and promoted FLS proliferation (Fig. 3C), suggesting that FoxO1 is involved in SMYD5 mediated FLS proliferation. To further confirm the regulatory effect of SMYD5 on FLS proliferation, we conducted a complementary experiment by blocking FoxO1 upon SMYD5 knockdown, and found that the inhibition of FLS proliferation and inflammatory response caused by SMYD5 gene silencing was reversed by FoxO1 knockdown (Fig. 3D, E). Taken together, these results suggest that SMYD5-mediated FLS proliferation depends on FoxO1.

Since SMYD5 primarily catalyzes H4K20 trimethylation and inhibits transcription, we examined the expression of H4K20me3 and the level of *Foxo1* mRNA. Unfortunately, intervention targeting SMYD5 did not alter H4K20me3 levels or regulate FoxO1 transcription (Fig. S2A–D), indicating that SMYD5-mediated inhibition of FoxO1 expression might depend on posttranslational modifications. Next, we utilized cycloheximide (CHX), a protein synthesis inhibitor, to assess FoxO1 stability. We observed that FoxO1 stability diminished with SMYD5 overexpression (Fig. 3F), yet it persisted for up to 6 h when SMYD5 was knocked down (Fig. 3G). It has been reported that protein ubiquitination is involved in FoxO1 degradation, so we used MG132, a proteasome inhibitor, to probe the degradation pathway. The reduction in FoxO1 protein level caused by CHX was markedly recovered by MG132 with or without IL-1β (Fig. 3H), indicating that FoxO1 is indeed degraded via ubiquitin–proteasome pathway. We further exogenously transfected HEK293T with His-SMYD5 and HA-FoxO1, and then conducted co-IP assay. We observed that SMYD5 coprecipitated with FoxO1 (Fig. 3I). Considering SMYD5 is a histone methyltransferase and FoxO1 protein is strictly regulated by modifications on its protein [23], we hypothesized that SMYD5 could methylate FoxO1. As expected, cotransfection of SMYD5 and FoxO1 in HEK293T cells significantly increased the lysine methylation level of FoxO1 protein (Fig. 3J). Furthermore, the in vitro methylation experiment confirmed that SMYD5 could promote the methylation of FoxO1 in a dose-dependent manner (Fig. 3K). Following this, we next tried to figure out whether SMYD5-mediated methylation affected FoxO1 stability, and found that FoxO1 polyubiquitylation was markedly increased in the presence of SMYD5 (Fig. 3L). Collectively, the above results suggest that SMYD5 promotes FLS proliferation by methylating FoxO1 and enhancing its ubiquitination-mediated degradation.

### SMYD5 promotes inflammatory response by activating NF-κB signaling pathway

**Fig. 4:**
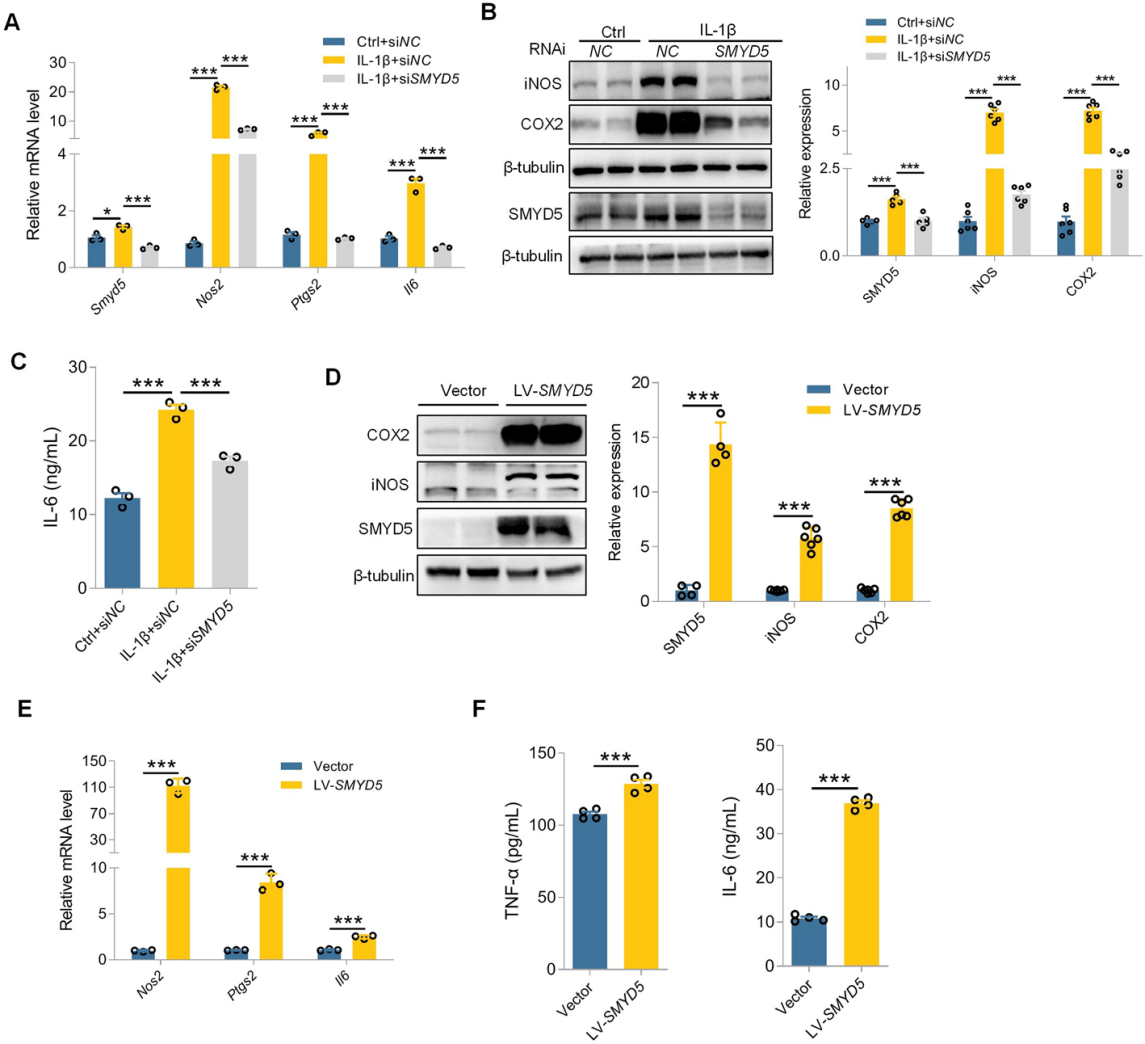
SMYD5 promotes inflammatory response in IL-1β-induced FLS. **A–C** SMYD5 was knocked down using *SMYD5* siRNA in IL-1β (10 ng/ml, 24 h)-induced FLS, with control siRNA (si*NC*) as the negative control. **A** RT-qPCR analysis of *Smyd5*, *Nos2*, *Ptgs2*, and *Il6* mRNA levels, *n* = 3. **B** Immunoblot analysis and quantification of SMYD5, iNOS, and COX2, with β-tubulin as a loading control, *n* = 5 or *n* = 6. **C** ELISA analysis for IL-6 in the supernatant from IL-1β induced FLS pretransfected with or without *SMYD5* siRNA, *n* = 3. **D**–**F** SMYD5 was overexpressed in FLS using lentivirus vectors encoding SMYD5 (*LV-SMYD5*). **D** Immunoblot analysis and quantification of SMYD5, iNOS, and COX2, with β-tubulin as a loading control, *n* = 4 or *n* = 6. **E** RT-qPCR analysis of *Nos2*, *Ptgs2*, and *Il6* mRNA levels, *n* = 3. **F** ELISA for IL-6 and TNF-α levels in the supernatant of FLS with or without LV-*SMYD5* treatment, *n* = 4. Data presented as mean ± SEM, *p* values calculated by two-tailed Student’s *t*-test (**D**–**F**) or one-way ANOVA test (**A**–**C**), *p < 0.05, ***p < 0.001

Inflammation is another key phenotypic change that occurs in FLS. Subsequent research mainly focused on whether SMYD5 regulates the inflammatory response of FLS. As expected, SMYD5 gene silencing significantly inhibited the upregulation of protein expression and gene transcription of inflammatory factors in IL-1β-induced FLS, such as iNOS (*Nos2*), COX2 (*Ptgs2*), and *Il6* (Fig. 4A, B), as well as reduced IL-6 secretion in the supernatant under IL-1β stimulation (Fig. 4C). We further explored whether SMYD5 alone could drive inflammatory response in FLS in the absence of IL-1β, and found that SMYD5 overexpression not only enhances the gene transcription and protein expression of aforementioned inflammatory factors but also boosts the secretion of IL-6 and TNF-α (Fig. 4D–F). These data indicate a significant positive correlation between SMYD5 expression and the inflammatory response in IL-1β-induced FLS.

**Fig. 5:**
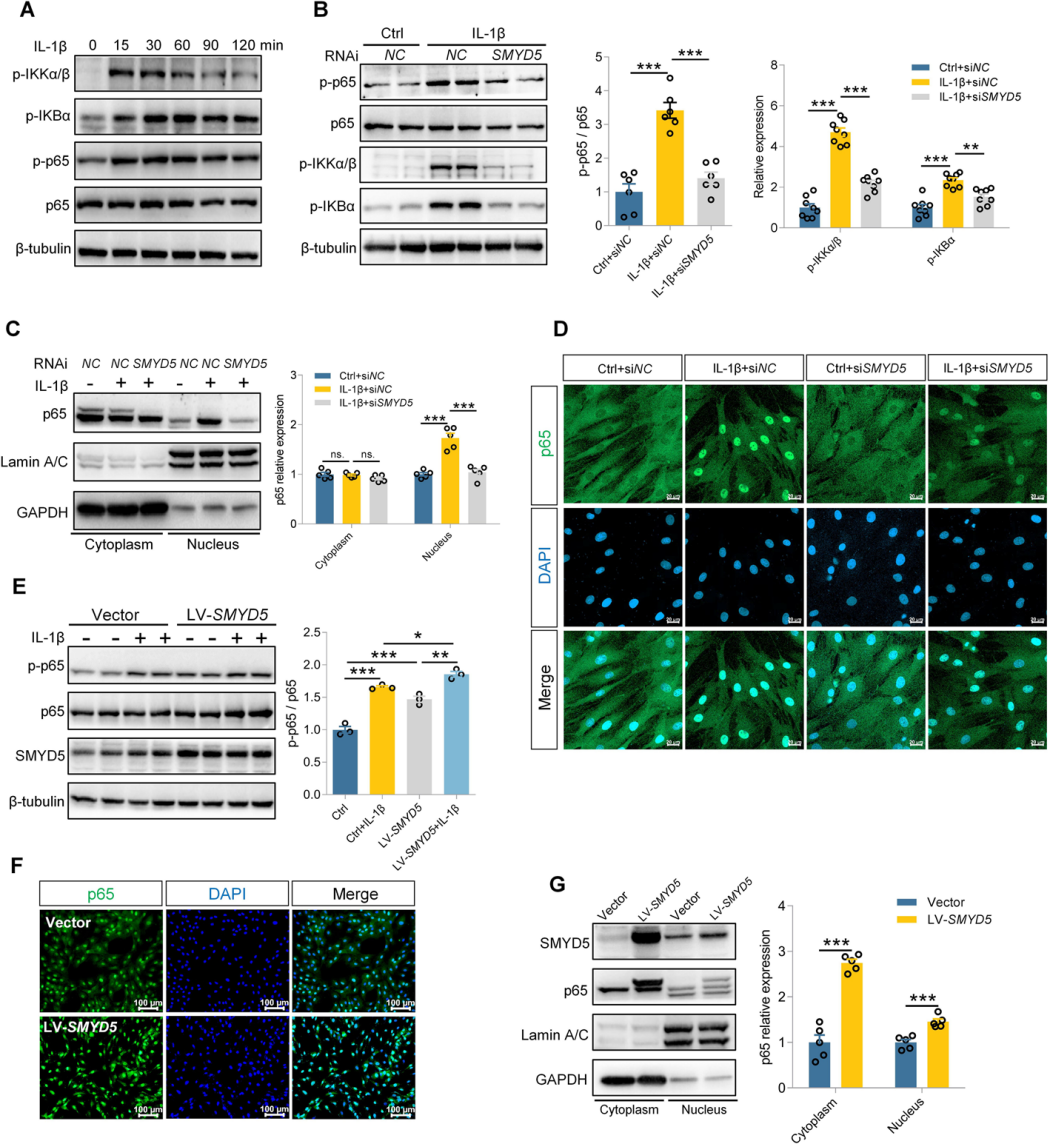
SMYD5 promotes inflammatory response by activating NF-κB signaling pathway. **A** FLS were treated with IL-1β (10 ng/ml) for indicated times, and cell lysates were analyzed using phosphor-specific antibodies for p65, p-IKKα/β, and p-IKBα to assess NF-κB signaling activation. (**B**–**D**) FLS were pretransfected with si*NC* or si*SMYD5* and then incubated with 10 ng/ml IL-1β for 15 min. **B** Immunoblot analysis of whole-cell lysates for expression and phosphorylation of key NF-κB subunits. Right panel: quantification of p65 phosphorylation (*n* = 6) and p-IKKα/β and p-IKBα expression (*n* = 8). **C** Immunoblot analysis of nuclear and cytoplasmic p65 distribution. p65 expression was normalized to Lamin A/C (nuclear) or GAPDH (cytoplasmic), *n* = 5. **D** Immunofluorescence staining to assess nuclear translocation of p65. Scale bars, 50 μm. **E**–**G** FLS pretransfected with or without LV-*SMYD5* were treated with 10 ng/ml IL-1β for 15 min. **E** Immunoblot analysis of p65 phosphorylation. p-p65 expression was normalized to total p65, *n* = 3. **F** Immunofluorescence staining to examine p65 distribution between nuclear and cytoplasmic compartments. Scale bars, 100 μm. **G** Immunoblot analysis of nuclear and cytoplasmic p65 distribution. p65 expression was normalized to Lamin A/C (nuclear) or GAPDH (cytoplasmic), *n* = 5. Data presented as mean ± SEM, ***p < 0.001, at least three independent experiments were repeated. Data presented as mean ± SEM, *p*-Values were calculated by two-tailed Student’s *t*-test (**G**) or one-way ANOVA test (**B**, **C**, **E**), *p < 0.05, **p < 0.01, ***p < 0.001, n.s. means no significance

Upon activation, nuclear factor-kappa B (NF-κB) translocates into the nucleus and promotes the transcription of downstream inflammatory factors such as *Nos2* and *Ptgs2*, playing a crucial role in RA progression [24]. We found that IL-1β could activate NF-κB by promoting p65 phosphorylation (Fig. 5A). Notably, SMYD5 knockdown dramatically reduced the p65 phosphorylation (Fig. 5B) and nuclear translocation (Fig. 5C), consistent with the immunofluorescence staining results (Fig. 5D). Conversely, overexpression of SMYD5 alone activated the NF-κB signaling pathway to a degree nearly equivalent to IL-1β treatment. When combined with IL-1β stimulation, it further increased the p65 phosphorylation (Fig. 5E). Additionally, SMYD5 overexpression enhanced the nuclear accumulation of p65 (Fig. 5F, G). Collectively, these findings indicate that SMYD5 can promote the FLS inflammatory response via NF-κB signaling pathway.

### SMYD5-mediated inflammatory response depends on upregulation of HK2

**Fig. 6:**
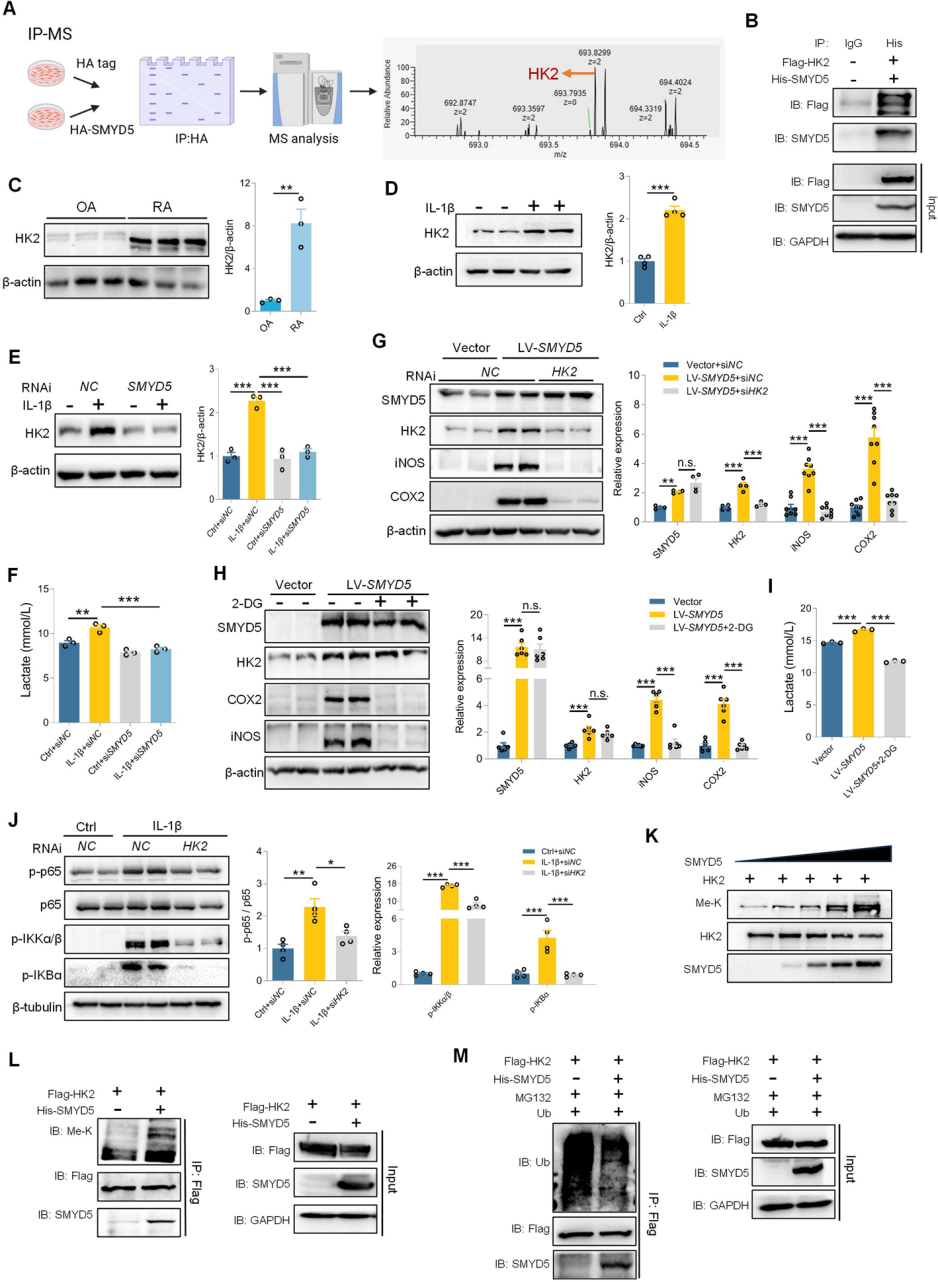
SMYD5-mediated inflammatory response depends on upregulation of HK2. **A** IP-MS was performed to identify SMYD5-interacting proteins. **B** HEK293T cells were transfected with His-SMYD5 and Flag-HK2 plasmids, followed by co-IP using anti-His antibody. **C** Immunoblot analysis of HK2 in synovial tissues from patients with RA or OA; HK2 levels were quantified relative to β-actin, *n* = 3. **D** FLS were incubated with IL-1β. Immunoblot analysis and quantification of HK2 are shown with β-actin as a loading control, *n* = 3. **E** FLS transfected with control or *SMYD5* siRNA were incubated with or without IL-1β. Immunoblot analysis and quantification of HK2 are shown with β-actin as a loading control, *n* = 3. **F** Lactate levels in the supernatant were measured using a lactate detection kit, *n* = 3. **G**–**I** FLS infected with LV-*SMYD5* were challenged with *HK2* siRNA (**G**) or 10 mM 2-DG (**H**). Immunoblot of SMYD5, HK2, iNOS, and COX2 was quantified relative to β-actin (**G**, *n* = 4 or *n* = 8; **H**, *n* = 6). **I** Lactate levels in the supernatant were measured, *n* = 3. **J** FLS pretransfected with control or *HK2* siRNA were treated with IL-1β. Immunoblot analysis detected expression and phosphorylation of NF-κB subunits. Phosphorylation of p65, p-IKKα/β, and p-IκBα was quantified, *n* = 4. **K** In vitro methyltransferase assay: recombinant HK2 was incubated with SMYD5 and analyzed by SDS-PAGE. **L** HEK293T cells transfected with Flag-HK2 and His-SMYD5 were immunoprecipitated with anti-Flag and probed for HK2 methylation. **M** HEK293T cells were cotransfected, and co-IP showed decreased HK2 polyubiquitylation in the presence of SMYD5. Data presented as mean ± SEM, *p* values calculated by two-tailed Student’s *t*-test (**C**) or one-way ANOVA test (**D**–**I**), *p < 0.05, **p < 0.01, ***p < 0.001, n.s. means no significance

To fully understand the mechanism by which SMYD5 regulates RA inflammation, we overexpressed SMYD5 in FLS and performed immunoprecipitation and mass spectrometry analysis to unbiasedly identify the proteins that interact with SMYD5. Surprisingly, we found an interaction between HK2 and SMYD5 (Fig. 6A), which was validated in HEK293T cells via the co-IP assay (Fig. 6B). HK2 is the first key enzyme that catalyzes glycolysis. It has been reported that increased expression of HK2 in RA-FLS can promote the development of RA [25], which is supported by our experiments. We observed elevated HK2 expression in the synovium tissues of patients with RA (Fig. 6C) and in IL-1β-induced FLS (Fig. 6D). As expected, knocking down HK2 in FLS could reduce IL-1β-induced inflammatory response (Fig. S3). To further explore the regulatory effect of SMYD5 on HK2, we knocked down SMYD5 using *SMYD5* siRNA and found a significant decrease in HK2 expression in IL-1β-induced FLS (Fig. 6E). Lactate is a key metabolite of glycolysis; therefore, we measured the lactate level in the supernatant of FLS, and found that IL-1β induced an increase in lactate release, whereas knocking down SMYD5 inhibited lactate accumulation (Fig. 6F). Furthermore, overexpression of SMYD5 in FLS significantly enhanced the expression of HK2 and inflammatory mediators, as well as increased lactate accumulation. This effect is partially counteracted by *HK2* siRNA or 2-DG (a competitive inhibitor of hexokinases) (Fig. 6G–I). Notably, neither HK2 gene silencing nor its inhibition impacted SMYD5 expression, suggesting that HK2 functions downstream of SMYD5 (Fig. 6G, H). Since SMYD5 upregulation can mediate NF-κB signaling pathway, we aimed to investigate whether HK2 plays a role in activating this pathway. To explore this, we knocked down HK2 and then stimulated FLS with IL-1β for 15 min. We observed inhibition of NF-κB signaling pathway following HK2 knockdown (Fig. 6J). Previous reports indicate that lactate induces inflammatory responses in FLS through the NF-κB pathway [26], and our findings further support the viewpoint that lactate may participate in HK2-mediated activation of NF-κB signaling pathway in FLS.

The above data confirmed that the SMYD5–HK2–NF-κB signaling axis plays a crucial role in the inflammatory response of FLS. However, the mechanism by which SMYD5 regulates HK2 remains unclear. In vitro methylation experiment demonstrated that SMYD5 could methylate HK2 in a dose-dependent manner (Fig. 6K). Consistent with this result, it was found that SMYD5 could methylate HK2 when His-SMYD5 and Flag-HK2 were ectopically expressed in HEK293T cells (Fig. 6L). Notably, the ectopic expression of SMYD5 significantly inhibited the ubiquitination degradation of HK2 (Fig. 6M). On the basis of these findings, we concluded that SMYD5 upregulates HK2 by methylating it and reducing its ubiquitination, thereby promoting lactate release and activating NF-κB signaling pathway, ultimately leading to an inflammatory response.

### Intraarticular delivery of AAV-sh*SMYD5* alleviates arthritis severity in CIA mice

**Fig. 7:**
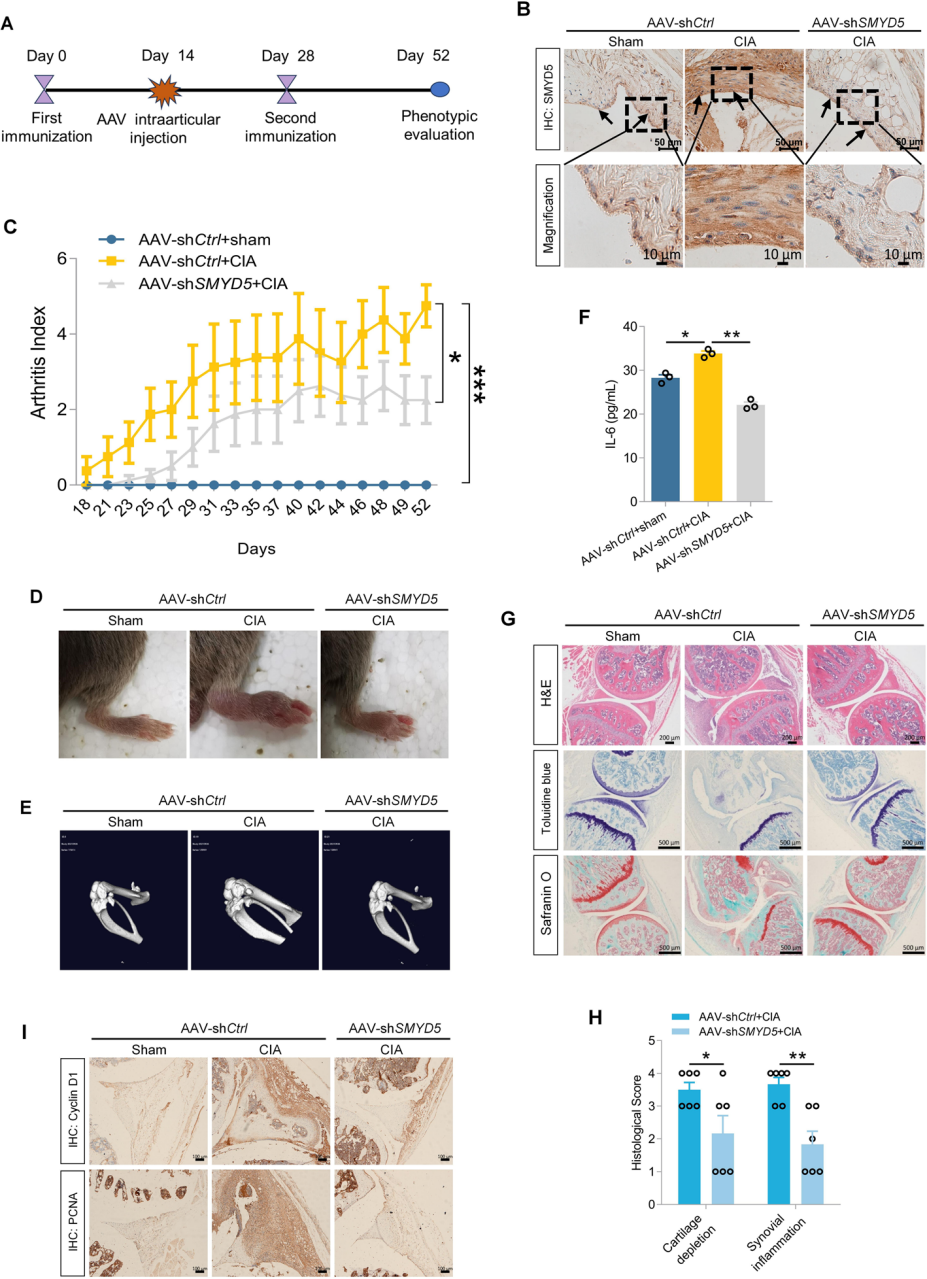
Intraarticular delivery of AAV-sh*SMYD5* alleviates arthritis severity in CIA mice. **A** Schematic of the experimental design. Mice were immunized with CII and randomly assigned to receive intraarticular injections of 10 μl AAV-sh*Ctrl* or AAV-sh*SMYD5* on day 14. **B** Immunohistochemical staining of SMYD5 in the synovium of mice. Scale bars, 10 μm. **C** SMYD5 knockdown delayed disease onset and alleviated the arthritis score in CIA mice, *n* = 8. **D** Representative images of CIA mice ankles. Erythema and swelling were markedly observed in the CIA mice, while AAV-sh*SMYD5* treatment significantly alleviated the arthritis severity. **E** Micro-CT revealed significant reduction in bone and cartilage damage in the joints of CIA mice treated with AAV-sh*SMYD5*. **F** ELISA analysis of IL-6 levels in mice serum on day 25 post-second immunization, *n* = 3. **G** Representative ankle joint images stained with hematoxylin and eosin (H&E), Toluidine Blue, and Safranin O. **H** Quantification of synovial inflammation and cartilage depletion based on H&E and Toluidine Blue/Safranin O staining, *n* = 6. **I** Immunohistochemical staining of Cyclin D1 and PCNA in synovial tissues of each group. Data presented as mean ± SEM, *p* values calculated by two-tailed Student’s *t*-test (**H**) or one-way ANOVA test (**C**, **F**), *p < 0.05, **p < 0.01, ***p < 0.001

The CIA mice model shares many pathological features with human RA, including synovial hyperplasia, joint swelling, and cartilage destruction [27], which can be established in genetically susceptible DBA/1J mice immunized with bovine type II collagen (CII) emulsified in complete Freund’s adjuvant (CFA). Given the potential role of SMYD5 in mediating proliferation and inflammation in FLS, we next validated these effects in CIA mice model via injecting adeno-associated virus vectors carrying SMYD5 short-hairpin RNA (AAV-sh*SMYD5*) into knee joints to knock down SMYD5; the schedule is shown in Fig. 7A. As expected, increased expression of SMYD5 was found in the synovium of immunized mice, while intraarticular injection of AAV-sh*SMYD5* successfully reduced SMYD5 expression compared with mice injected with AAV control shRNA (AAV-sh*Ctrl*) (Fig. 7B). According to the Arthritis Index, SMYD5 knockdown delayed the onset and alleviated the severity of arthritis in CIA mice (Fig. 7C). In line with this, representative images of mice’s hind paws also demonstrated that SMYD5 knockdown improved hind paw thickness and swelling in CIA mice (Fig. 7D). In addition, micro-CT showed a significant reduction in bone and cartilage damage in the joints treated with AAV-sh*SMYD5* (Fig. 7E). CIA mice were sacrificed on day 52 for further examination. The level of IL-6 in serum of CIA mice was increased, while knocking down SMYD5 resulted in a decrease in IL-6 secretion (Fig. 7F). Histological analysis revealed that SMYD5 knockdown protected CIA mice from severe synovial inflammation and synovial hyperplasia. Safranin O and Toluidine Blue staining showed that cartilage injury induced by collagen immunity was significantly alleviated after AAV-sh*SMYD5* injection (Fig. 7G, H). Immunohistochemical staining also indicated that AAV-sh*SMYD5* caused a dramatic decrease in the expression of Cyclin D1 and PCNA (Fig. 7I). Overall, these data suggest that SMYD5 knockdown has protective effects on synovial hyperplasia, joint inflammation, and cartilage destruction in CIA mice.

## Discussion

Inflammation and abnormal proliferation of FLS are typical pathological features of RA. Intervention strategies targeting FLS proliferation and inflammation offer a new approach to treating RA, as they can be implemented without immune consequences. Epigenetic modifications play an important role in joint damage by epigenetically imprinting FLS in RA. Here, we observed that SMYD5, an H4K20 methyltransferase, was prominently expressed in synovial tissues of patients with RA and IL-1β-treated FLS. We showed that SMYD5 mediated the methylation of FoxO1 and HK2 to promote FLS proliferation and inflammation in RA pathogenesis. Importantly, administering AAV-sh*SMYD5* directly into the joints of CIA mice substantially reduced the severity of arthritis by decreasing joint inflammation, cartilage damage, and bone loss.

SMYD5 belongs to the SMYDs family, and studies on SMYD5 have primarily focused on self-renewal and differentiation of embryonic stem cells. The correlation between SMYD5 and inflammatory response has been discussed in macrophages, where SMYD5, functioning as a component of NCoR corepressor complexes, controlled proinflammatory gene programs by modulating the TLR4 promoter’s H4K20me3 modification [13]. In the current study, we confirmed that SMYD5 was abundantly expressed both in the synovium of patients with RA and in FLS induced by IL-1β. In response to immune microenvironment, activated FLS exhibit a consecutively higher proliferation rate and insufficient apoptotic ability, accompanied by the release of various pro-inflammatory mediators and tissue-degenerative enzymes such as MMPs, promoting inflammation and joint destruction [28, 29]. Our further study showed that the prominent expression of SMYD5 could promote the proliferation and inflammation in FLS, while knockdown of SMYD5 improved the aforementioned IL-1β-induced-FLS phenotype, suggesting that SMYD5 may be a potential therapeutic target for RA treatment.

FoxO1, a transcription factor, plays a crucial role in regulating the cell cycle and proliferation, thus contributing to the development of RA [22, 30]. Studies have shown that the expression of FoxO1 in the synovium is negatively correlated with FLS proliferation and inflammation [22]. Consistently, our preliminary findings from IL-1β-treated FLS demonstrated a decrease in FoxO1 levels, accompanied by increased proliferation. Notably, silencing SMYD5 led to a significant recovery of FoxO1 expression, which, in turn, effectively suppressed FLS proliferation and the inflammatory response triggered by IL-1β. This finding suggested that FoxO1 is a key mediator of the RA-FLS-like phenotype regulated by SMYD5. The opposite changes between FoxO1 and RA-FLS-like phenotype may correlate to transcriptional inactivation, marked by SMYD5 or H4K20me3. However, silencing or overexpressing SMYD5 failed to directly affect FoxO1 mRNA level in IL-1β-stimulated FLS, indicating the involvement of alternative regulatory mechanisms. Besides transcriptional regulation, FoxO1 has also been widely reported to be regulated by methylation, acetylation, ubiquitination, and other posttranslational mechanisms [31]. For instance, the degradation of the FoxO1 protein through G9a-catalyzed methylation can boost cell proliferation and worsen colon cancer [32]. In an IBD study, SMYD5 was identified as a key factor in exacerbating intestinal inflammation and injury through a non-histone methylation-dependent manner [16]. Encouragingly, we came to a similar conclusion in IL-1β-induced FLS that SMYD5 enhances FoxO1’s ubiquitination and degradation by methylating it, thereby promoting FLS proliferation.

RA-FLS undergo metabolic changes and transform into a tumor cell-like phenotype characterized by accelerated glycolysis, gluconeogenesis, or the pentose phosphate pathway. These processes aim to meet the increasing energy demands associated with FLS proliferation, invasive migration, and inflammatory responses [33–35], while glucose deprivation significantly improves RA-FLS-like phenotype [36]. Hexokinases (HKs) are key enzymes that catalyze the first step of glucose metabolism, converting glucose into glucose 6-phosphate (G6P) and initiating subsequent steps in glucose utilization. The evidence that HK2 selective overexpression in the inflammatory synovium of RA rather than in normal tissues suggests that HK2 can serve as a metabolic target for treating RA without compromising whole-body glucose homeostasis [25]. HK2 inhibitors, such as 2-DG, have been widely studied and shown to counteract inflammation, proliferation, and invasion in RA-FLS, thereby reducing the severity of arthritis [2]. Consistent with these findings, our results indicated that SMYD5 could exacerbate RA-FLS inflammation in an HK2-dependent manner, wherein SMYD5 exerted positive regulation on HK2 through crosstalk of nonhistone methylation and ubiquitination. Furthermore, pharmacological inhibition of HK2 could mitigate the upregulation of inflammatory genes induced by SMYD5 overexpression. Previous studies have shown that HK2 plays a pro-inflammatory role by activating the NF-κB signaling pathway in neuroinflammatory diseases related to microglial activation [37]. Besides, the expression of HK2 has been positively correlated with extracellular lactate accumulation in RA-FLS [25], which could further activate NF-κB signaling transduction and lead to FLS inflammatory response [38]. Our results supported this viewpoint and demonstrated the important regulatory role of the SMYD5–HK2–NF-κB signaling cascade in RA-FLS inflammation. Moreover, our results also suggested that lactate may be involved in this process. Notably, SMYD5 has been reported as a negative regulator of inflammatory genes in macrophages [13], arguing against our findings in FLS. This difference could be attributed to SMYD5 performing different functions by methylating histone or nonhistone proteins, respectively.

Our study suggested that SMYD5 plays a functionally critical role in the pathogenesis of RA. In current mainstream RA treatments, long-term and high-dose systemic administration achieves a sustained therapeutic concentration in affected joints but also increases the risk of adverse reactions, including off-target organ toxicity [39, 40]. Additionally, although RA is a systemic disease, some studies have indicated that joint inflammation tends to recur in the same joints during the RA disease course [41]. In recent years, various intraarticular drug delivery strategies, particularly with the AAV vector system, have emerged, which makes the combination of systemic and local treatment a promising therapeutic strategy [42, 43]. It has been reported that intraarticular delivery of AAV vectors encoding PD-L1 attenuates joint inflammation and tissue damage in a mouse model of RA [42]. Compared with systemic administration of drugs through nonspecific routes, intraarticular drug intervention specifically targets the affected joint cavity, achieving precise treatment of RA at a lower drug dose and frequency, and avoids the systemic toxicity and immunosuppression [44]. It has been reported that one-time delivery of genes to the synovium using AAV vectors can induce sustained therapeutic gene expression locally in the joints and maintain stability for up to 10 years [45]. Although intraarticular gene therapy using AAV vectors has been considered a feasible treatment option for RA, and many clinical trials targeting a single cytokine such as IL-6 and TNF-α have been conducted [46, 47], the clinical benefits remain limited. Therefore, there is an urgent need for effective potential targets. According to data from the International Mouse Phenotyping Consortium, SMYD5 knockout does not affect mice fertility, and the overall phenotype of the mice remains largely normal. Thus, we hypothesize that the systemic consequences of SMYD5 knockout or inhibition are minimal, making it a promising candidate for combination therapy. On the basis of these considerations and the lack of a specific inhibitor for SMYD5, we chose to deliver *SMYD5* shRNA into both the left and right knee joints using AAV vectors to knock down SMYD5. Compared with the AAV-control group, the AAV-sh*SMYD5* group exhibited reduced SMYD5 expression, decreased cartilage damage, and less joint swelling. Notably, local AAV-sh*SMYD5* injection also lowered serum IL-6 levels and alleviated swelling in the mouse toe joints, despite the virus not being directly injected into these sites. Given that the joint cavity is a relatively enclosed space, this observation was unexpected. It was reported that local adenovirus-mediated gene transfer of viral IL-10 can have an anti-arthritic effect on distant, untreated joints [48]. Similar observations have been made in other studies [49, 50], though the underlying mechanisms remain unclear. Additionally, the possibility of viral leakage into the circulatory system cannot be excluded. This phenomenon warrants further investigation in future studies. Overall, these findings suggest that intraarticular administration of AAV-sh*SMYD5* may be a promising therapeutic approach for RA.

## Conclusions

This study identified SMYD5 as a novel regulator for synovial injury in RA, which was supported by two lines of evidence: first, the SMYD5–FoxO1 signaling axis drives FLS proliferation; second, through the SMYD5–HK2–NF-κB pathway, FLS are activated to produce numerous inflammatory mediators. These findings suggest that targeting SMYD5 could offer a novel therapeutic approach for RA beyond conventional immunotherapies. Furthermore, we demonstrated that a single intraarticular injection of AAV-sh*SMYD5* could induce robust gene silencing and relieve joint symptoms, highlighting the advantage of local treatment of RA. Further studies focusing on optimizing the transduction efficiency and maintaining the long-term efficiency of AAV-sh*SMYD5* vectors will lay a foundation for potential clinical applications of SMYD5-based therapies.

## Supplementary Information

Additional file 1.

## Data Availability

All data generated or analyzed during this study are included in this published article and its supplementary information files.

## Abbreviations

RA: Rheumatoid arthritis
FLS: Fibroblast-like synoviocytes
ECM: Extracellular matrix
MMPs: Matrix metalloproteinases
SMYDs: SET and MYND domain containing proteins
IBD: Inflammatory bowel disease
siRNA: Small interfering RNA
CIA: Collagen-induced arthritis
CFA: Complete Freund’s adjuvant
IFA: Incomplete Freund’s adjuvant
CII: Bovine type II collagen
AAV: Adeno-associated virus
AAV-sh*SMYD5*: AAV vectors carrying SMYD5 short-hairpin RNA
AAV-sh*Ctrl*: AAV vectors carrying negative control short-hairpin RNA
DMEM: Dulbecco’s modified Eagle’s medium
FBS: Fetal bovine serum
ELISA: Enzyme-linked immunosorbent assay
co-IP: Co-immunoprecipitation
H&E: Hematoxylin and eosin
H4K20: Histone H4 lysine 20
FoxO1: Forkhead box protein O1
HKs: Hexokinases
2-DG: 2-Deoxyglucose
IF: Immunofluorescence
IHC: Immunohistochemical
NF-κB: Nuclear factor-kappa B
OA: Osteoarthritis
CHX: Cycloheximide
IACUC: Institutional Animal Care and Use Committee
SPF: Specific pathogen-free
IL-1β: Interleukin-1 beta
SDS-PAGE: Sodium dodecyl sulfate–polyacrylamide gel electrophoresis

## References

[1] Smolen JS, Aletaha D, McInnes IB. Rheumatoid arthritis. Lancet. 2016;388:2023–38. 10.1016/S0140-6736(16)30173-8. 27156434 10.1016/S0140-6736(16)30173-8

[2] Nemeth T, Nagy G, Pap T. Synovial fibroblasts as potential drug targets in rheumatoid arthritis, where do we stand and where shall we go? Ann Rheum Dis. 2022;81:1055–64. 10.1136/annrheumdis-2021-222021. 35715191 10.1136/annrheumdis-2021-222021 PMC9279838

[3] Smolen JS, Aletaha D, Barton A, Burmester GR, Emery P, Firestein GS, et al. Rheumatoid arthritis. Nat Rev Dis Primers. 2018;4:18001. 10.1038/nrdp.2018.1. 29417936 10.1038/nrdp.2018.1

[4] Nagy G, Roodenrijs NMT, Welsing PMJ, Kedves M, Hamar A, van der Goes MC, et al. EULAR points to consider for the management of difficult-to-treat rheumatoid arthritis. Ann Rheum Dis. 2022;81:20–33. 10.1136/annrheumdis-2021-220973. 34407926 10.1136/annrheumdis-2021-220973 PMC8761998

[5] Bartok B, Firestein GS. Fibroblast-like synoviocytes: key effector cells in rheumatoid arthritis. Immunol Rev. 2010;233:233–55. 10.1111/j.0105-2896.2009.00859.x. 20193003 10.1111/j.0105-2896.2009.00859.x PMC2913689

[6] Filer A. The fibroblast as a therapeutic target in rheumatoid arthritis. Curr Opin Pharmacol. 2013;13:413–9. 10.1016/j.coph.2013.02.006. 23562164 10.1016/j.coph.2013.02.006

[7] Firestein GS. Biomedicine. Every joint has a silver lining. Science. 2007;315:952–3. 10.1126/science.1139574. 17303744 10.1126/science.1139574

[8] Bottini N, Firestein GS. Duality of fibroblast-like synoviocytes in RA: passive responders and imprinted aggressors. Nat Rev Rheumatol. 2013;9:24–33. 10.1038/nrrheum.2012.190. 23147896 10.1038/nrrheum.2012.190 PMC3970924

[9] Klein K, Gay S. Epigenetics in rheumatoid arthritis. Curr Opin Rheumatol. 2015;27:76–82. 10.1097/BOR.0000000000000128. 25415526 10.1097/BOR.0000000000000128

[10] Liu Y, Aryee MJ, Padyukov L, Fallin MD, Hesselberg E, Runarsson A, et al. Epigenome-wide association data implicate DNA methylation as an intermediary of genetic risk in rheumatoid arthritis. Nat Biotechnol. 2013;31:142–7. 10.1038/nbt.2487. 23334450 10.1038/nbt.2487 PMC3598632

[11] Nakano K, Whitaker JW, Boyle DL, Wang W, Firestein GS. DNA methylome signature in rheumatoid arthritis. Ann Rheum Dis. 2013;72:110–7. 10.1136/annrheumdis-2012-201526. 22736089 10.1136/annrheumdis-2012-201526 PMC3549371

[12] Karami J, Aslani S, Tahmasebi MN, Mousavi MJ, Sharafat Vaziri A, Jamshidi A, et al. Epigenetics in rheumatoid arthritis; fibroblast-like synoviocytes as an emerging paradigm in the pathogenesis of the disease. Immunol Cell Biol. 2020;98:171–86. 10.1111/imcb.12311. 31856314 10.1111/imcb.12311

[13] Stender JD, Pascual G, Liu W, Kaikkonen MU, Do K, Spann NJ, et al. Control of proinflammatory gene programs by regulated trimethylation and demethylation of histone H4K20. Mol Cell. 2012;48:28–38. 10.1016/j.molcel.2012.07.020. 22921934 10.1016/j.molcel.2012.07.020 PMC3472359

[14] Kidder BL, Hu G, Cui K, Zhao K. SMYD5 regulates H4K20me3-marked heterochromatin to safeguard ES cell self-renewal and prevent spurious differentiation. Epigenetics Chromatin. 2017;10:8. 10.1186/s13072-017-0115-7. 28250819 10.1186/s13072-017-0115-7 PMC5324308

[15] Kidder BL, He R, Wangsa D, Padilla-Nash HM, Bernardo MM, Sheng S, et al. SMYD5 controls heterochromatin and chromosome integrity during embryonic stem cell differentiation. Cancer Res. 2017;77:6729–45. 10.1158/0008-5472.CAN-17-0828. 28951459 10.1158/0008-5472.CAN-17-0828 PMC5804482

[16] Hou Y, Sun X, Gheinani PT, Guan X, Sharma S, Zhou Y, et al. Epithelial SMYD5 exaggerates IBD by down-regulating mitochondrial functions via post-translational control of PGC-1alpha stability. Cell Mol Gastroenterol Hepatol. 2022;14:375–403. 10.1016/j.jcmgh.2022.05.006. 35643234 10.1016/j.jcmgh.2022.05.006 PMC9249919

[17] Wu W, Wang J, Xiao C, Su Z, Su H, Zhong W, et al. SMYD2-mediated TRAF2 methylation promotes the NF-kappaB signaling pathways in inflammatory diseases. Clin Transl Med. 2021;11: e591. 10.1002/ctm2.591. 34841684 10.1002/ctm2.591 PMC8567046

[18] Wu W J, Wang J H, Xiao C X, Su Z H, Su H B, Zhong W, et al. SMYD2-mediated TRAF2 methylation promotes the NF-κB signaling pathways in inflammatory diseases. Clin Transl Med. 2021;(11). 10.1002/ctm2.591. 10.1002/ctm2.591 PMC8567046 34841684

[19] Jia W, Wu W, Yang D, Xiao C, Su Z, Huang Z, et al. Histone demethylase JMJD3 regulates fibroblast-like synoviocyte-mediated proliferation and joint destruction in rheumatoid arthritis. FASEB J. 2018;32:4031–42. 10.1096/fj.201701483R. 29481307 10.1096/fj.201701483R

[20] Svensson MND, Zoccheddu M, Yang S, Nygaard G, Secchi C, Doody KM, et al. Synoviocyte-targeted therapy synergizes with TNF inhibition in arthritis reversal. Sci Adv. 2020;6: eaba4353. 10.1126/sciadv.aba4353. 32637608 10.1126/sciadv.aba4353 PMC7319753

[21] Feng J, Ding C, Qiu N, Ni X, Zhan D, Liu W, et al. Firmiana: towards a one-stop proteomic cloud platform for data processing and analysis. Nat Biotechnol. 2017;35:409–12. 10.1038/nbt.3825. 28486446 10.1038/nbt.3825

[22] Grabiec AM, Angiolilli C, Hartkamp LM, van Baarsen LG, Tak PP, Reedquist KA. JNK-dependent downregulation of FoxO1 is required to promote the survival of fibroblast-like synoviocytes in rheumatoid arthritis. Ann Rheum Dis. 2015;74:1763–71. 10.1136/annrheumdis-2013-203610. 24812285 10.1136/annrheumdis-2013-203610

[23] Yamagata K, Daitoku H, Takahashi Y, Namiki K, Hisatake K, Kako K, et al. Arginine methylation of FOXO transcription factors inhibits their phosphorylation by Akt. Mol Cell. 2008;32:221–31. 10.1016/j.molcel.2008.09.013. 18951090 10.1016/j.molcel.2008.09.013

[24] Sendo S, Machado CRL, Boyle DL, Benschop RJ, Perumal NB, Choi E, et al. Dysregulated NUB1 and neddylation enhances rheumatoid arthritis fibroblast-like synoviocyte inflammatory responses. Arthritis Rheumatol. 2024. 10.1002/art.42856. 38566346 10.1002/art.42856

[25] Bustamante MF, Oliveira PG, Garcia-Carbonell R, Croft AP, Smith JM, Serrano RL, et al. Hexokinase 2 as a novel selective metabolic target for rheumatoid arthritis. Ann Rheum Dis. 2018;77:1636–43. 10.1136/annrheumdis-2018-213103. 30061164 10.1136/annrheumdis-2018-213103 PMC6328432

[26] Zou Y, Zeng S, Huang M, Qiu Q, Xiao Y, Shi M, et al. Inhibition of 6-phosphofructo-2-kinase suppresses fibroblast-like synoviocytes-mediated synovial inflammation and joint destruction in rheumatoid arthritis. Br J Pharmacol. 2017;174:893–908. 10.1111/bph.13762. 28239846 10.1111/bph.13762 PMC5386999

[27] Brand DD, Latham KA, Rosloniec EF. Collagen-induced arthritis. Nat Protoc. 2007;2:1269–75. 10.1038/nprot.2007.173. 17546023 10.1038/nprot.2007.173

[28] Matsuda K, Shiba N, Hiraoka K. New insights into the role of synovial fibroblasts leading to joint destruction in rheumatoid arthritis. Int J Mol Sci. 2023;24:5173. 10.3390/ijms24065173. 36982247 10.3390/ijms24065173 PMC10049180

[29] Yan M, Komatsu N, Muro R, Huynh NC, Tomofuji Y, Okada Y, et al. ETS1 governs pathological tissue-remodeling programs in disease-associated fibroblasts. Nat Immunol. 2022;23:1330–41. 10.1038/s41590-022-01285-0. 35999392 10.1038/s41590-022-01285-0

[30] van der Vos KE, Coffer PJ. The extending network of FOXO transcriptional target genes. Antioxid Redox Signal. 2011;14:579–92. 10.1089/ars.2010.3419. 20673124 10.1089/ars.2010.3419

[31] Eijkelenboom A, Burgering BM. FOXOs: signalling integrators for homeostasis maintenance. Nat Rev Mol Cell Biol. 2013;14:83–97. 10.1038/nrm3507. 23325358 10.1038/nrm3507

[32] Chae YC, Kim JY, Park JW, Kim KB, Oh H, Lee KH, et al. FOXO1 degradation via G9a-mediated methylation promotes cell proliferation in colon cancer. Nucleic Acids Res. 2019;47:1692–705. 10.1093/nar/gky1230. 30535125 10.1093/nar/gky1230 PMC6393239

[33] Ahn JK, Kim S, Hwang J, Kim J, Kim KH, Cha HS. GC/TOF-MS-based metabolomic profiling in cultured fibroblast-like synoviocytes from rheumatoid arthritis. Joint Bone Spine. 2016;83:707–13. 10.1016/j.jbspin.2015.11.009. 27133762 10.1016/j.jbspin.2015.11.009

[34] Wei K, Korsunsky I, Marshall JL, Gao A, Watts GFM, Major T, et al. Notch signalling drives synovial fibroblast identity and arthritis pathology. Nature. 2020;582:259–64. 10.1038/s41586-020-2222-z. 32499639 10.1038/s41586-020-2222-z PMC7841716

[35] Biniecka M, Canavan M, McGarry T, Gao W, McCormick J, Cregan S, et al. Dysregulated bioenergetics: a key regulator of joint inflammation. Ann Rheum Dis. 2016;75:2192–200. 10.1136/annrheumdis-2015-208476. 27013493 10.1136/annrheumdis-2015-208476 PMC5136702

[36] Jose AM. New potential therapeutic approaches targeting synovial fibroblasts in rheumatoid arthritis. Biochem Pharmacol. 2021;194: 114815. 10.1016/j.bcp.2021.114815. 34715065 10.1016/j.bcp.2021.114815

[37] Cheng J, Zhang R, Xu Z, Ke Y, Sun R, Yang H, et al. Early glycolytic reprogramming controls microglial inflammatory activation. J Neuroinflammation. 2021;18:129. 10.1186/s12974-021-02187-y. 34107997 10.1186/s12974-021-02187-y PMC8191212

[38] Manosalva C, Quiroga J, Hidalgo AI, Alarcon P, Anseoleaga N, Hidalgo MA, et al. Role of lactate in inflammatory processes: friend or foe. Front Immunol. 2021;12: 808799. 10.3389/fimmu.2021.808799. 35095895 10.3389/fimmu.2021.808799 PMC8795514

[39] Guo Q, Wang Y, Xu D, Nossent J, Pavlos NJ, Xu J. Rheumatoid arthritis: pathological mechanisms and modern pharmacologic therapies. Bone Res. 2018;6:15. 10.1038/s41413-018-0016-9. 29736302 10.1038/s41413-018-0016-9 PMC5920070

[40] Butoescu N, Jordan O, Doelker E. Intra-articular drug delivery systems for the treatment of rheumatic diseases: a review of the factors influencing their performance. Eur J Pharm Biopharm. 2009;73:205–18. 10.1016/j.ejpb.2009.06.009. 19545624 10.1016/j.ejpb.2009.06.009

[41] Heckert SL, Bergstra SA, Matthijssen XME, Goekoop-Ruiterman YPM, Fodili F, Ten Wolde S, et al. Joint inflammation tends to recur in the same joints during the rheumatoid arthritis disease course. Ann Rheum Dis. 2022;81:169–74. 10.1136/annrheumdis-2021-220882. 34462262 10.1136/annrheumdis-2021-220882

[42] Li W, Sun J, Feng SL, Wang F, Miao MZ, Wu EY, et al. Intra-articular delivery of AAV vectors encoding PD-L1 attenuates joint inflammation and tissue damage in a mouse model of rheumatoid arthritis. Front Immunol. 2023;14:1116084. 10.3389/fimmu.2023.1116084. 36936967 10.3389/fimmu.2023.1116084 PMC10021025

[43] Deviatkin AA, Vakulenko YA, Akhmadishina LV, Tarasov VV, Beloukhova MI, Zamyatnin Jr AA, et al. Emerging concepts and challenges in rheumatoid arthritis gene therapy. Biomedicines. 2020;8:9. 10.3390/biomedicines8010009. 31936504 10.3390/biomedicines8010009 PMC7168286

[44] Burt HM, Tsallas A, Gilchrist S, Liang LS. Intra-articular drug delivery systems: overcoming the shortcomings of joint disease therapy. Expert Opin Drug Deliv. 2009;6:17–26. 10.1517/17425240802647259. 19236205 10.1517/17425240802647259

[45] Kuzmin DA, Shutova MV, Johnston NR, Smith OP, Fedorin VV, Kukushkin YS, et al. The clinical landscape for AAV gene therapies. Nat Rev Drug Discov. 2021;20:173–4. 10.1038/d41573-021-00017-7. 33495615 10.1038/d41573-021-00017-7

[46] Evans CH, Ghivizzani SC, Robbins PD. Gene delivery to joints by intra-articular injection. Hum Gene Ther. 2018;29:2–14. 10.1089/hum.2017.181. 29160173 10.1089/hum.2017.181 PMC5773261

[47] Zavvar M, Assadiasl S, Soleimanifar N, Pakdel FD, Abdolmohammadi K, Fatahi Y, et al. Gene therapy in rheumatoid arthritis: strategies to select therapeutic genes. J Cell Physiol. 2019;234:16913–24. 10.1002/jcp.28392. 30809802 10.1002/jcp.28392

[48] Lechman ER, Keravala A, Nash J, Kim SH, Mi Z, Robbins PD. The contralateral effect conferred by intra-articular adenovirus-mediated gene transfer of viral IL-10 is specific to the immunizing antigen. Gene Ther. 2003;10:2029–35. 10.1038/sj.gt.3302109. 14566362 10.1038/sj.gt.3302109

[49] Ghivizzani SC, Lechman ER, Kang R, Tio C, Kolls J, Evans CH, et al. Direct adenovirus-mediated gene transfer of interleukin 1 and tumor necrosis factor alpha soluble receptors to rabbit knees with experimental arthritis has local and distal anti-arthritic effects. Proc Natl Acad Sci USA. 1998;95:4613–8. 10.1073/pnas.95.8.4613. 9539786 10.1073/pnas.95.8.4613 PMC22538

[50] Adriaansen J, Vervoordeldonk MJ, Tak PP. Gene therapy as a therapeutic approach for the treatment of rheumatoid arthritis: innovative vectors and therapeutic genes. Rheumatology (Oxford). 2006;45:656–68. 10.1093/rheumatology/kel047. 16510530 10.1093/rheumatology/kel047

