# Supplementary material for: Novel regulation mechanism of histone methyltransferase SMYD5 in rheumatoid arthritis
Source: Cell Mol Biol Lett. 2025 Mar 31;30:38. doi: 10.1186/s11658-025-00707-9 (PMC11959843; doi:10.1186/s11658-025-00707-9)
Supplement: Supplementary file 1 — Additional file 1. [file 11658_2025_707_MOESM1_ESM.docx]

**Additional file 1**

**
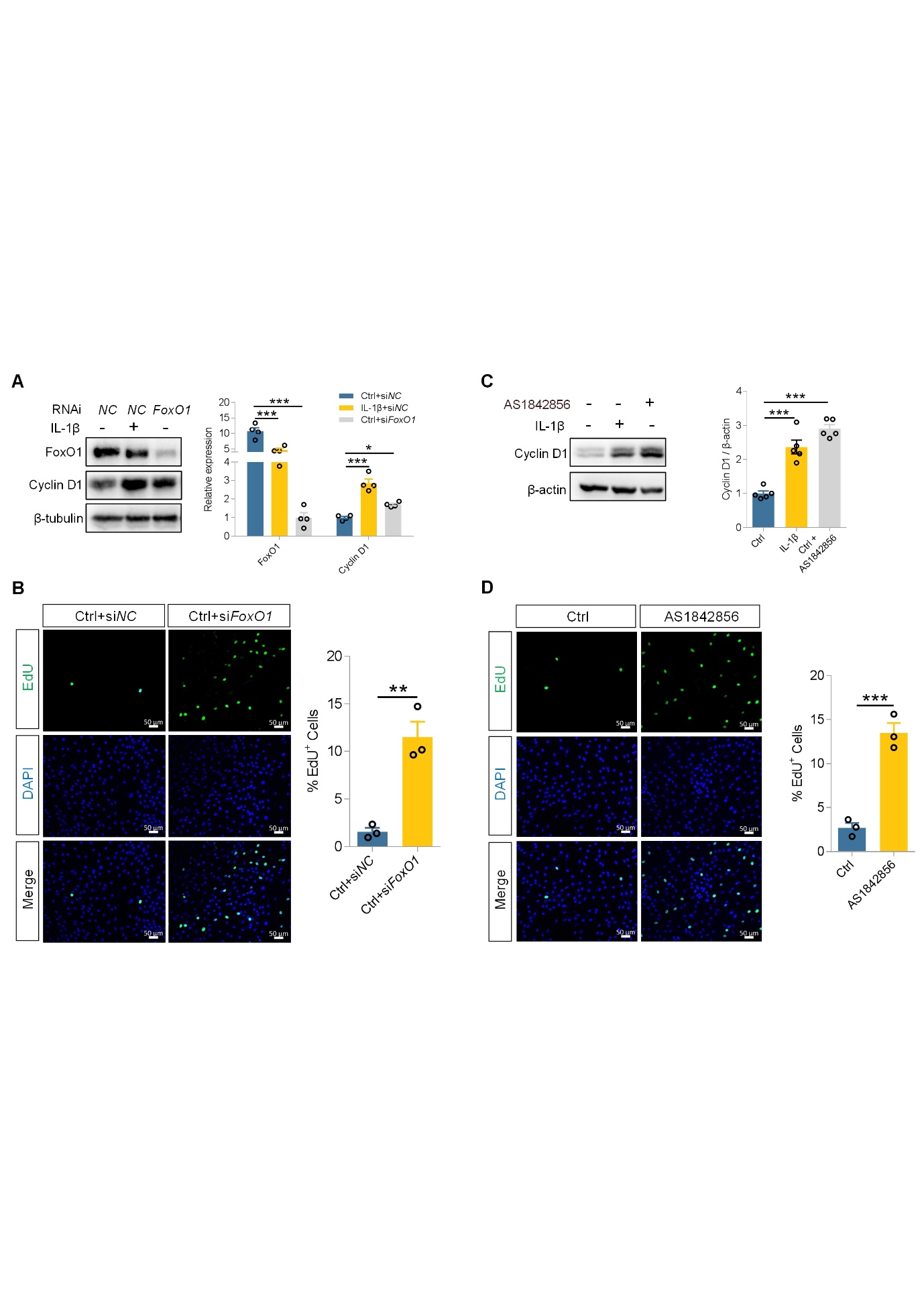
**

**Figure S1. Knockdown or inhibition of FoxO1 promotes FLS proliferation.** **(A)** Immunoblot analysis and quantification of FoxO1 and Cyclin D1 in FLS transfected with *control* or *FoxO1* siRNA, n=4. **(B)** EdU staining and quantification of the proportion of EdU^+^ cells in FLS transfected with *control* or *FoxO1* siRNA. Scale bars, 50 μm, n=3. **(C)** Immunoblots analysis and quantification of Cyclin D1 expression in FLS treated with or without AS1842856, n=5. **(D)** EdU staining and quantification of the proportion of EdU^+^ cells in FLS treated with or without AS1842856. Scale bars, 50 μm, n=3. Data presented as mean ± SEM, *p* values calculated by two-tailed Student’s t-test **(B, D)** or one-way ANOVA test **(A, C)**, ^*^*p* < 0.05, ^**^*p* < 0.01, ^***^*p* < 0.001.

**
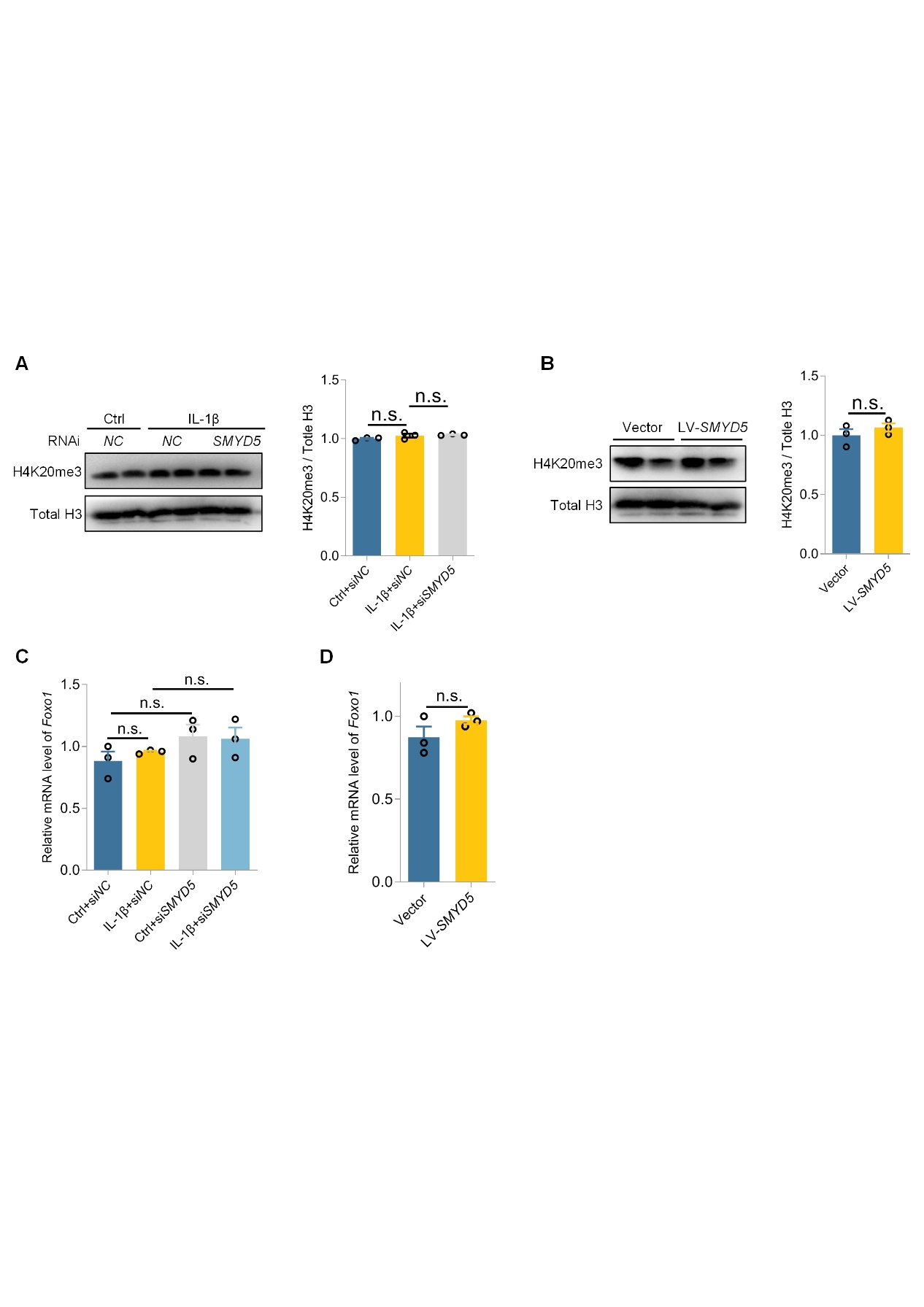
**

**Figure S2. SMYD5 knockdown or overexpression does not affect H4K20me3 expression or *Foxo1* transcription.** SMYD5 was knocked down using siRNA or overexpressed using LV-*SMYD5,* followed by incubation with or without IL-1β. **(A-B)** Immunoblot analysis and quantification for H4K20me3, n=3. **(C-D)** RT-qPCR analysis of *Foxo1* mRNA level, n=3. Data presented as mean ± SEM, *p* values calculated by two-tailed Student’s t-test **(B, D)** or one-way ANOVA test **(A, C)**, n.s. means no significance.


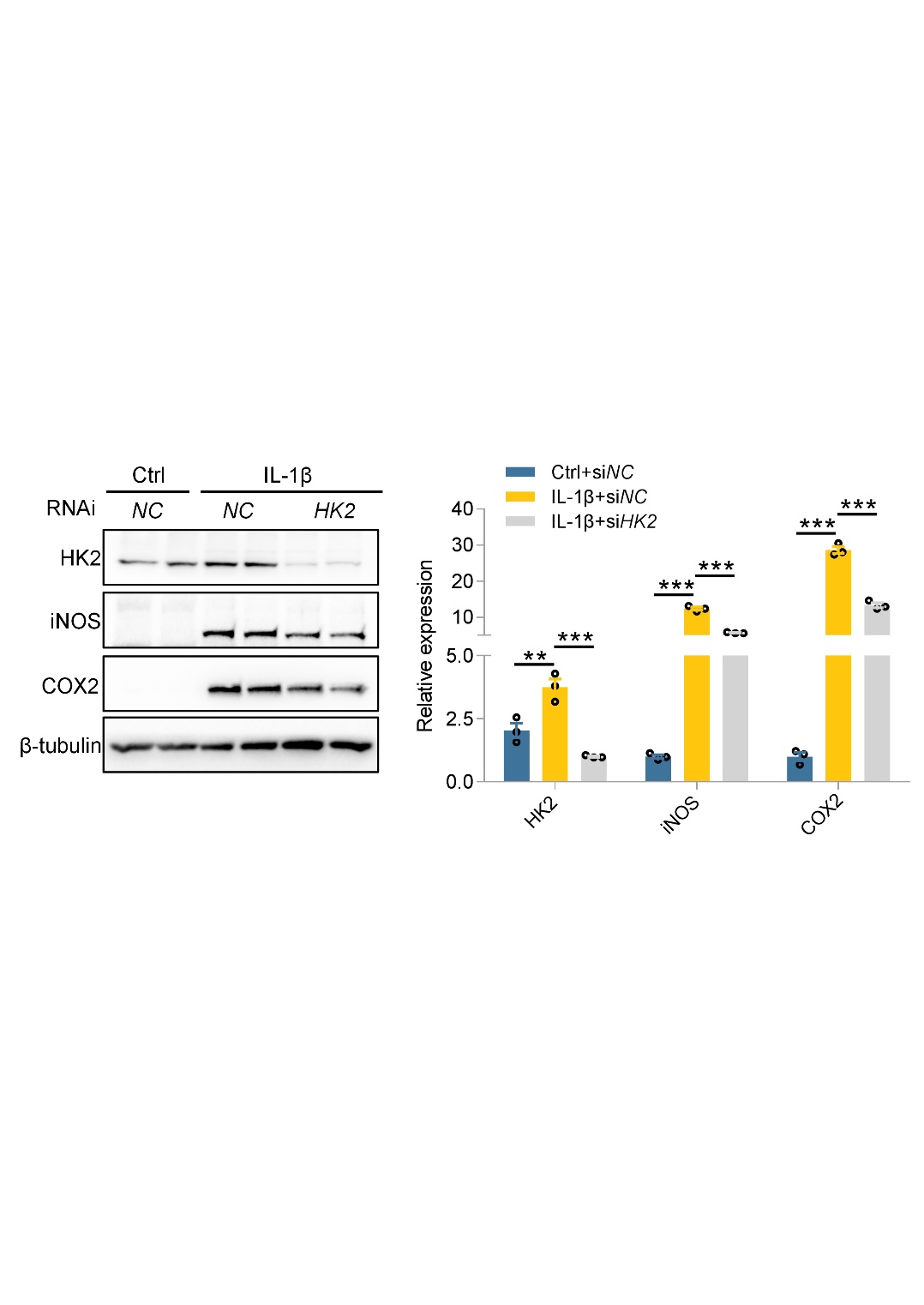


**Figure S3.** **HK2 knockdown inhibits inflammatory response in IL-1β induced FLS.** SMYD5 was knocked down using *HK2* siRNA in IL-1β (10 ng/ml, 24 h)-induced FLS, with *control* siRNA (si*NC*) as a negative control. Immunoblot analysis and quantification of HK2, iNOS and COX2 are shown, with β-tubulin as a loading control, n=3. Data presented as mean ± SEM, *p* values calculated by one-way ANOVA test, ^**^*p* < 0.01, ^***^*p* < 0.001.

**Supplementary Table S1**

**Antibodies**

| **Target antigen** | **Vendor or Source** | **Catalog #** | **Working concentration** | **Application** |
| --- | --- | --- | --- | --- |
| β-Actin | Proteintech | 66009-1-Ig | 0.1 μg/ml | WB |
| β-tubulin | Proteintech | 66240-1-Ig | 0.1 μg/ml | WB |
| GAPDH | Proteintech | 60004-1-Ig | 0.1 μg/ml | WB |
| iNOS | Proteintech | 18985-1-AP | 1:1000 | WB |
| Flag tag | Proteintech | 66008-4-Ig | 1:5000 | WB |
| His tag | Proteintech | 66005-1-Ig | 1:5000 | WB |
| HA tag | ShareBio | SB-AB0004 | 1:1000 | WB |
| SMYD5 | Absci | AB38742 | 1:1000 | WB |
| COX-2 | ABclonal | A1253 | 1:1000 | WB |
| HK2 | Cell Signaling Technology | #2867 | 1:1000 | WB |
| FoxO1 | Cell Signaling Technology | #2880 | 1:1000 | WB |
| p65 | Cell Signaling Technology | #8242T | 1:1000 | WB |
| p-p65 | Cell Signaling Technology | #3031S | 1:1000 | WB |
| p-IκBα | Cell Signaling Technology | #2859T | 1:1000 | WB |
| p-IKKα/β | Cell Signaling Technology | #2697T | 1:1000 | WB |
| Histone H3 | Cell Signaling Technology | #4499 | 1:2000 | WB |
| H4K20me3 | Abcam | ab177190 | 1:1000 | WB |
| Cyclin D1 | Abcam | ab134175 | 1:5000 | WB |
| MMP9 | Servicebio Technology | GB12132 | 1:1000 | WB |
| Mono/Di-Methyllysine | PTM BIO | PTM-602 | 1:1000 | WB |
| Lamin A/C | Santa Cruz Biotechnology | sc-3762 | 1:1000 | WB |
| p65 | Cell Signaling Technology | 8242T | 1:1000 | IF |
| OB-cadherin | Santa Cruz Biotechnology | sc-365867 | 1:200 | IF |
| SMYD5 | Absci | AB38742 | 1:100 | IF |
| SMYD5 | Absci | AB38742 | 1:100 | IHC |
| Cyclin D1 | Abcam | ab134175 | 1:250 | IHC |
| PCNA | Abcam | ab92552 | 1:250 | IHC |

**Supplementary Table S2**

Arthritis Index

| **Score** | **Symptom description** |
| --- | --- |
| 0 | No symptoms of arthritis |
| 1 | Swelling or redness of 1-2 toe joints |
| 2 | Swelling or redness of 1-2 toe joints or redness of one large joint |
| 3 | More than 4 joints redness and swelling |
| 4 | Severe arthritis, involving the entire foot |

**Supplementary Table S3**

Histological score (Cartilage depletion)

| **Score** | **Symptom description** |
| --- | --- |
| 0 | no cartilage destruction |
| 1 | localized cartilage erosions |
| 2 | more extended cartilage erosions |
| 3 | severe cartilage erosions |
| 4 | depletion of entire cartilage |

Histological Score (Synovial inflammation)

| **Score** | **Symptom description** |
| --- | --- |
| 0 | normal |
| 1 | minimal infiltration of inflammatory cells in periarticular area |
| 2 | mild infiltration |
| 3 | moderate infiltration |
| 4 | marked infiltration |

**Supplementary Table S4**

The sequences for siRNA

| **Clone Name** | **Sequence（5‘-3‘）** | | **Source / Repository** |
| --- | --- | --- | --- |
| Rat *SMYD5* siRNA | Sense | GAGGAGUGUUCACUAUCCUTT | GenePharma |
|  | Antisense | AGGAUAGUGAACACUCCUCTT |  |
| Rat *FoxO1* siRNA | Sense | GCACCGACUUUAUGAGCAATT | GenePharma |
|  | Antisense | UUGCUCAUAAAGUCGGUGCTT |  |
| Rat *HK2* siRNA | Sense | CCAUCCCACAGGAGGUUAUTT | GenePharma |
|  | Antisense | AUAACCUCCUGUGGGAUGGTT |  |
| Rat *Ctrl* siRNA | Sense | UUCUCCGAACGUGUCACGUTT | GenePharma |
|  | Antisense | ACGUGACACGUUCGGAGAATT |  |

**Supplementary Table S5**

The primers sequences for RT-qPCR

| Name | Sequence |
| --- | --- |
| Rat_*Nos2*_F | CACAGAGGGCTCAAAGGAGG |
| Rat_*Nos2*_R | AAAGTGGTAGCCACATCCCG |
| Rat_*Ptgs2*_F | CTCAGCCATGCAGCAAATCC |
| Rat_*Ptgs2*_R | GGGTGGGCTTCAGCAGTAAT |
| Rat_*Il6*_F | CTCTCCGCAAGAGACTTCCA |
| Rat_*Il6*_R | CTCCTCTCCGGACTTGTGAA |
| Rat_*Smyd5*_F | CCAACGGTCAAGGGATTGGA |
| Rat_*Smyd5*_R | AAGGAACTCTCCGGTTGCTG |
| Rat_*Foxo1*_F | TAACTGTGCCCCAGGACTCT |
| Rat_*Foxo1*_R | GATGTGTGAGGCATGGTGTTC |
| Rat_*Ccnd1*_F | CAAGTGTGACCCGGACTGC |
| Rat_*Ccnd1*_R | GACCAGCTTCTTCCTCCACTT |
| Rat_*Actb*_F | TGAGGGTTACGCGCTCC |
| Rat_*Actb*_R | CATCGGAACCGCTCATTGC |

**Supplementary Table S6**

**Clinical data for RA and OA patients**

| Characteristic | OA1 | OA2 | RA1 | RA2 |
| --- | --- | --- | --- | --- |
| Age (years) | 60 | 58 | 50 | 52 |
| Gender | female | female | female | female |
| Disease duration (years) | 3 | 2 | 1 | 10 |
| SJC | 1 | 1 | 1 | 3 |
| TJC | 1 | 1 | 1 | 1 |
| ESR (mm/hr) | 16 | 17 | 30 | 6 |
| CRP (mg/L) | 1.5 | 1.8 | 28.7 | 0.5 |
| DAS28 score | 3.2 | 3.24 | 3.5 | 2.92 |
| Anti A-CCP (U/mL) | - | - | - | ＞200U/mL |
| Anti RF (IU/mL) | 5.7 | 36.2 | 270.9 | 15.1 |
| Medications |  |  |  |  |
| Corticosteroids | - | - | + | + |
| NSAIDs | + | + | + | + |
| DMARDs | - | - | + | + |
| Biologic agents | - | - | tocilizumab | - |

（SJC: Swollen joint count; TJC: Tender joint count; ESR: Erythrocyte sedimentation rate; CRP: C-reactive protein; DAS28 score: Disease Activity Score 28-joint count; Anti A-CCP: Anti-cyclic citrullinated peptide antibody; Anti RF: Anti-rheumatoid factor; NSAIDs: Non-steroidal anti-inflammatory drugs; DMARDs: Disease-modifying antirheumatic drugs）
